# Spatial Heterogeneity in Particle‐Associated, Light‐Independent Superoxide Production Within Productive Coastal Waters

**DOI:** 10.1029/2020JC016747

**Published:** 2020-10-16

**Authors:** Kevin M. Sutherland, Kalina C. Grabb, Jennifer S. Karolewski, Sydney Plummer, Gabriela A. Farfan, Scott D. Wankel, Julia M. Diaz, Carl H. Lamborg, Colleen M. Hansel

**Affiliations:** ^1^ Department of Marine Chemistry and Geochemistry Woods Hole Oceanographic Institution Woods Hole MA USA; ^2^ Department of Earth, Atmospheric and Planetary Science Massachusetts Institute of Technology Cambridge MA USA; ^3^ Now at Department of Earth and Planetary Science Harvard University Cambridge MA USA; ^4^ Skidaway Institute of Oceanography, Department of Marine Sciences University of Georgia Savannah GA USA; ^5^ Now at Scripps Institution of Oceanography University of California, San Diego La Jolla CA USA; ^6^ Department of Mineral Sciences Smithsonian Institution Washington DC USA; ^7^ Ocean Sciences Department University of California Santa Cruz CA USA

**Keywords:** reactive oxygen species, extracellular superoxide, light‐independent ROS

## Abstract

In the marine environment, the reactive oxygen species (ROS) superoxide is produced through a diverse array of light‐dependent and light‐independent reactions, the latter of which is thought to be primarily controlled by microorganisms. Marine superoxide production influences organic matter remineralization, metal redox cycling, and dissolved oxygen concentrations, yet the relative contributions of different sources to total superoxide production remain poorly constrained. Here we investigate the production, steady‐state concentration, and particle‐associated nature of light‐independent superoxide in productive waters off the northeast coast of North America. We find exceptionally high levels of light‐independent superoxide in the marine water column, with concentrations ranging from 10 pM to in excess of 2,000 pM. The highest superoxide concentrations were particle associated in surface seawater and in aphotic seawater collected meters off the seafloor. Filtration of seawater overlying the continental shelf lowered the light‐independent, steady‐state superoxide concentration by an average of 84%. We identify eukaryotic phytoplankton as the dominant particle‐associated source of superoxide to these coastal waters. We contrast these measurements with those collected at an off‐shelf station, where superoxide concentrations did not exceed 100 pM, and particles account for an average of 40% of the steady‐state superoxide concentration. This study demonstrates the primary role of particles in the production of superoxide in seawater overlying the continental shelf and highlights the importance of light‐independent, dissolved‐phase reactions in marine ROS production.

## Key Points

- Light‐independent superoxide concentrations exhibit significant variability in coastal waters along the northeast coast of North America
- Particles account for most light‐independent superoxide production in coastal water, but dissolved‐phase superoxide production also occurs
- Chlorophyll and eukaryotes are correlated with superoxide production rate, suggesting that eukaryotic phytoplankton are important ROS producers

## 1 Introduction

Reactive oxygen species (ROS) are short‐lived oxygen‐containing molecules with half‐lives in aquatic systems that range from fractions of seconds to days. The most common forms of ROS in marine systems include hydrogen peroxide (H_2_O_2_), superoxide (O_2_^•−^/HO_2_), hydroxyl radical (HO^•^), singlet oxygen (^1^O_2_), and carbonate radical (CO_3_^•−^), which are commonly found at picomolar to nanomolar levels. The formation of ROS within aqueous systems occurs via sequential one‐electron transfer reactions (Fridovich, 1998). For instance, the ROS O_2_^•−^, H_2_O_2_, and HO^•^ are the intermediates of the sequential reduction of molecular oxygen to water. Measurements of environmental ROS concentrations have focused primarily on hydrogen peroxide, which has a typical concentration range of nanomolar to micromolar, and secondarily on superoxide, which has a typical concentration range of picomolar to nanomolar. These ROS have been measured in a wide range of natural settings including marine (Hansard et al., 2010; Kieber et al., 2001; Roe et al., 2016; Rose et al., 2008b, 2010; Rusak et al., 2011; Yuan & Shiller, 2001, 2005; Zika et al., 1985), estuarine (Kieber & Helz, 1995; Szymczak & Waite, 1988; Zhang et al., 2016), and freshwater environments (Cooper & Lean, 1989; Cory et al., 2016; Richard et al., 2007; Zhang et al., 2016).

Historically, production of ROS within the surface ocean has been attributed solely to light‐induced abiotic reactions, particularly, excitation of colored dissolved organic matter or CDOM (Garg et al., 2011; Powers & Miller, 2014; Shaked & Rose, 2013). A number of key discoveries over the past decade have shown that biological processes, via both enzyme‐ and metabolite‐mediated pathways, are also important contributors to the production of the ROS superoxide and hydrogen peroxide in the surface ocean (Diaz et al., 2013, 2019; Hansard et al., 2010; Rose et al., 2008b; Rusak et al., 2011; Yuasa et al., 2020). Indeed, removal of particles that may be biotic and/or abiotic in nature from natural waters has been shown to significantly decrease superoxide and hydrogen peroxide production, demonstrating a presumable microbial origin of these ROS (Marsico et al., 2015; Roe et al., 2016; Zhang et al., 2016). Phytoplankton and heterotrophs both contribute to marine ROS production (Diaz et al., 2013; Sutherland et al., 2019), with phytoplankton in the surface ocean accounting for a significant proportion of marine superoxide production (Diaz & Plummer, 2018; Godrant et al., 2009; Rose et al., 2008b; Sutherland et al., 2020). Although the production of light‐independent, extracellular superoxide is ubiquitous among photosynthetic microorganisms, extracellular superoxide production by these organisms is enhanced by photosynthetically active radiation (PAR, Diaz et al., 2019; Plummer et al., 2019; Schneider et al., 2016; Yuasa et al., 2020). Multiple studies suggest that light‐mediated extracellular superoxide production in phototrophs is directly related to the accumulation of NADPH, implicating extracellular superoxide in maintaining redox homeostasis during photosynthesis (Diaz et al., 2019; Yuasa et al., 2020). The outer‐membrane enzyme that facilitates extracellular superoxide production in one particular group of diatoms has even been shown to persist after cell death (Diaz et al., 2019; Schneider et al., 2016). In the dark ocean, heterotrophic bacteria are thought to be the primary source of ROS. This includes representatives of the most abundant marine heterotroph group, SAR11 clade, which accounts for as much as a quarter of all cells in the ocean (Giovannoni, 2017; Sutherland et al., 2019). Concentrations of extracellular superoxide in dark seawater typically range from a few pM to as high as ~2,000 pM in surface waters (Hansard et al., 2010; Roe et al., 2016; Rose et al., 2008b; Rusak et al., 2011). Some productive near‐shore environments, including coral reefs, may exceed typical water column concentrations, with reported concentrations as high as ~100–200 nM (Diaz et al., 2016; Grabb et al., 2019).

The production, degradation, and steady‐state concentrations of superoxide play an important role in determining the abundance of other downstream ROS such as hydrogen peroxide. Superoxide dismutase (SOD) disproportionates superoxide to dioxygen and hydrogen peroxide at or near diffusion‐limited rates in natural waters (Fielden et al., 1974; Wolfe‐Simon et al., 2006). The typical lifetime of superoxide in natural water is on the order of a minute. Enzymatic elimination does not, however, have a monopoly on superoxide removal. Superoxide can be oxidized, reduced, and/or disproportionated by a wide variety of redox active metals and dissolved organic compounds (Wuttig et al., 2013b), many of which result in hydrogen peroxide formation.

The implications of ROS in the surface and deep ocean are far reaching. ROS play a key role in the remineralization of carbon and cycling of numerous metals within the ocean (Heller & Croot, 2010b; Rose, 2012; Wuttig et al., 2013a). For instance, superoxide is capable of oxidizing and/or reducing a number of metals, including copper (Cu), iodine (I), iron (Fe), and manganese (Mn) (Archibald & Fridovich, 1982; Hansard et al., 2011; Learman et al., 2013; Li et al., 2014; Rose, 2012; Voelker et al., 2000; Wuttig et al., 2013a). Importantly, superoxide also has the ability to reduce Fe, converting Fe (III) to Fe (II) (Rose, 2012; Voelker & Sedlak, 1995). Because Fe is an essential nutrient that limits photosynthesis in vast regions of the ocean, superoxide‐mediated reduction and subsequent release of Fe from strong Fe (III)‐ligands has been suggested as an important process controlling biological activity in the surface ocean (Rose et al., 2005). Hydrogen peroxide formed from superoxide dismutation is an oxidant of Fe (II) (Millero & Sotolongo, 1989; Moffett & Zika, 1987). This reaction forms the highly reactive hydroxyl radical, a ROS that rapidly degrades carbon, including recalcitrant forms such as lignin (Mopper & Zhou, 1990). ROS production associated with harmful algal blooms has been implicated in massive fish kills; however, the extent to which there is a causal link between ROS and ichthyotoxicity remains an open question (Dorantes‐Aranda et al., 2015; Kim et al., 1999; Tang et al., 2005). The widespread production of ROS in the dark and the light also underscores the many ways in which superoxide production can ultimately be a net sink of dissolved oxygen in the marine environment and thus influence the spatiotemporal availability of this dominant electron acceptor (Sutherland et al., 2020).

The physiological health and function of marine organisms are influenced by ROS levels in beneficial and detrimental ways. Superoxide has been implicated in oxidative stress in a broad range of organismal systems. However, the essential role of superoxide in the health and function of higher eukaryotes (animals, plants, and fungi) has long been appreciated (Aguirre et al., 2005; Lamb & Dixon, 1997). More recently, ROS have also been implicated in beneficial processes within microbes, including iron acquisition, cell signaling, redox homeostasis, and growth promotion in phytoplankton and heterotrophic bacteria (Buetler et al., 2004; Diaz et al., 2019; Hansel et al., 2019; Oda et al., 1995; Roe & Barbeau, 2014; Rose et al., 2008b; Saran, 2003).

It is therefore apparent that ROS have a complex and diverse role in the biogeochemistry and ecology of the ocean. In fact, the role of ROS in ocean health and function is receiving increasing attention, and the past decade has seen a surge of research exploring the importance of these compounds in marine productivity, biological health, and ocean chemistry (Bond et al., 2020; Diaz et al., 2019; Hansel et al., 2019; Hopwood et al., 2017; Rose, 2012; Yuasa et al., 2020). Nevertheless, the ROS concentrations and underlying formation processes have only been explored in a limited number of environmental systems. Here, we conducted the first study exploring the concentrations and dynamics of superoxide along the North Atlantic continental shelf off the East Coast of the United States. Our primary focus here is to understand how light‐independent superoxide concentrations change with the presence of particles in the marine water column and identify which environmental factors are most closely associated with superoxide dynamics in the marine water column.

## 2 Methods

### 2.1 Sample Location and Water Collection

**Figure 1:**
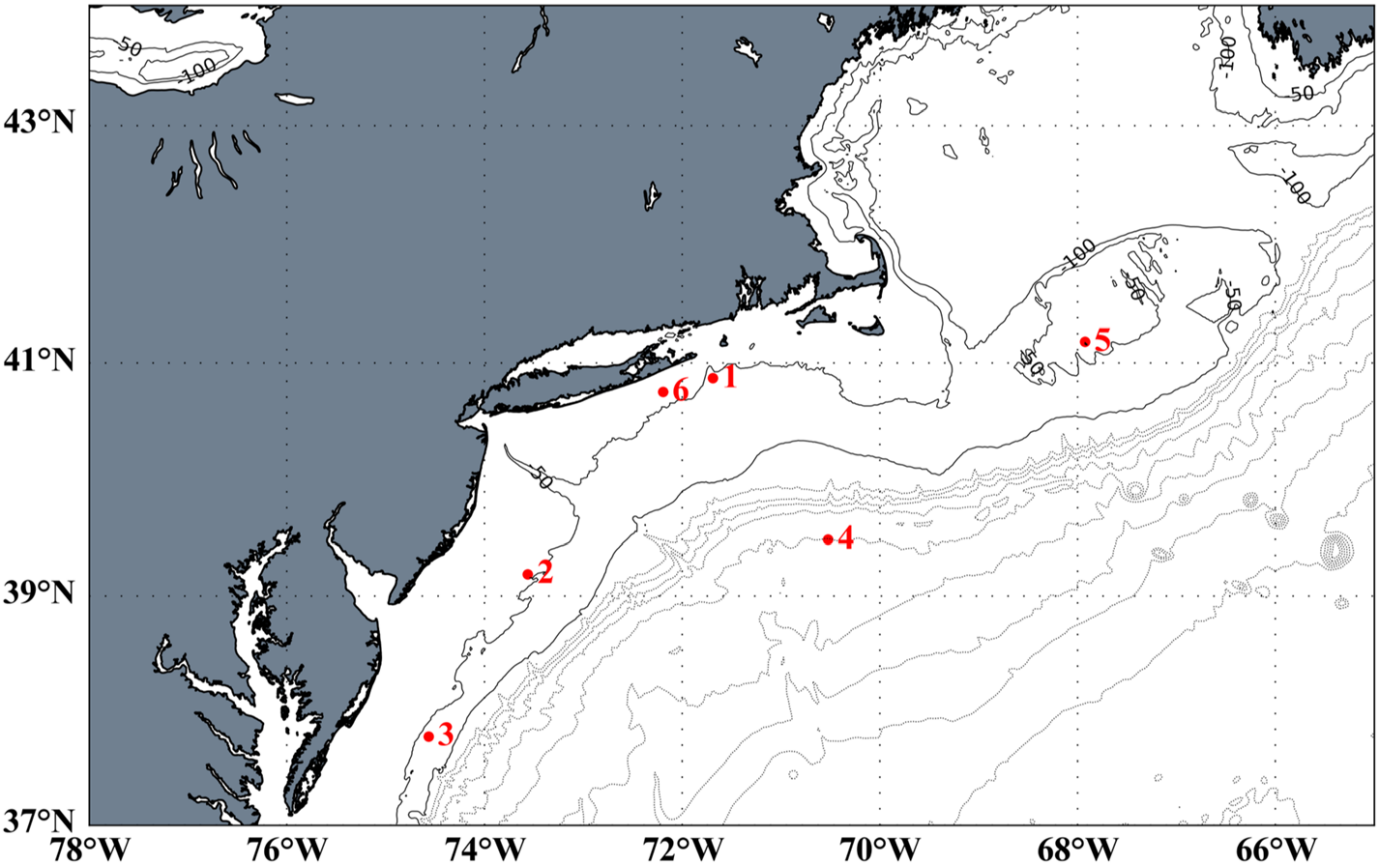
Sample locations of ROS measurements collected in this study in August 2017. Stations 1, 2, 3, 5, and 6 are on‐shelf sampling locations (max depth 50–60 m), and Station 4 is a deeper (max depth over 2,000 m) off‐shelf sampling location.

Water samples were collected from six water column sites along the East Coast of the United States (Figure 1 and Table S1 in the supporting information) in August 2017 aboard the *R/V Endeavor*. Five of the stations were located in waters overlying the continental shelf (Stations 1, 2, 3, 5, and 6), with typical water depths between 50 and 60 m. The remaining station, chosen as a contrast to the other five shelf sites, was collected off‐shelf in water exceeding 2,000 m depth (Station 4). Samples for ROS measurements were collected as previously described (Oldham et al., 2020). Briefly, water was collected using a paint‐sealed rosette, equipped with a Sea‐bird SBE 19 Plus V2 recording CTD and 8‐L X‐Niskins (General Oceanics). The Niskins were acid cleaned prior to deployment and soaked in local seawater for 12 hr prior to sample collection. This clean rosette was lowered on a nonmetallic line (1/4″ vinyl‐jacketed Vectran) with water column samples collected at preprogrammed depths controlled by a Sea‐bird Auto‐firing Module. Sample depths were chosen based on the water column hydrographic profiles collected just prior to launching the clean rosette. The water column profiling cast included measurement of dissolved oxygen, salinity, PAR, beam transmission, and fluorescence profiles (later converted to chlorophyll concentration with discrete sampling) reported here. Depths chosen were site specific and targeted the surface, the chlorophyll maximum, the photic‐aphotic transition, an aphotic depth between the photic‐aphotic transition and bottom, and 3 m above the sediment‐water interface. Here we defined the transition from photic to subphotic as the depth at which the PAR level reaches 1% of the surface value. Immediately following recovery of the clean rosette, the Niskins were removed from the frame and moved to a shipboard HEPA‐air filled “bubble,” where samples were immediately decanted through acid‐washed C‐flex silicone tubing directly into acid‐washed 1‐L PTFE bottles. Bottles were filled and rinsed with the seawater samples twice before filling the bottle with the sample to be measured. Samples were immediately analyzed for superoxide as follows.

### 2.2 Superoxide Measurements

#### 2.2.1 Superoxide Concentration

Water samples were collected directly from the clean rosette into acid‐washed, opaque plastic bottles and stored prior to analysis in a shipboard seawater incubator circulated continuously with site water from a depth of ~5 m. The temperature in the incubator, therefore, was that of the ambient seawater condition at the 5 m depth for that site (Figure S1). Water from each depth was given three separate treatments. The first treatment was unfiltered seawater (UFSW), which was incubated in the dark for 30 min to allow all light‐generated superoxide to decay. In the second treatment, seawater was filtered (0.2 μm), amended with 50 μM diethylene‐triaminepentaacetic acid (DTPA, Sigma), and aged in the dark for at least 8 hr (referred to as AFSW) in order to eliminate all particle‐ and metal‐associated superoxide (remaining sources would be reactive dissolved organic carbon (DOC) and/or soluble extracellular enzymes). In the third treatment, light‐independent, particle‐associated superoxide signals were determined by filtering (0.2 μm) a subset of UFSW samples approximately 30 min before analysis (termed fresh filtered seawater or FFSW). The total dark incubation periods of UFSW and FFSW were 30 min and 1 hr, respectively. The additional 30 min delay is necessary for FFSW because the act of filtration can temporarily increase superoxide concentrations (Roe et al., 2016). The 30 min delay (>10 half‐lives of superoxide) allows sufficient time for the superoxide concentration in FFSW to relax back to its dark, particle‐independent steady‐state concentration.

In the ship laboratory, superoxide signals were measured by pumping UFSW, FFSW, or AFSW from dark bottles using a high‐accuracy peristaltic pump directly into a flowthrough FeLume Mini system (Waterville Analytical, Waterville, ME). Superoxide detection was based on the reaction between superoxide and a chemiluminescent probe, a methyl cypridina luciferin analog (MCLA, Santa Cruz Biotechnology, Rose et al., 2008a) as described before (Roe et al., 2016). To minimize incidental room light exposure, samples were pumped into the FeLume using opaque tubing (approximately 20 s transit time between sample bottle and FeLume). For each depth, the superoxide signals were measured within UFSW, FFSW, and AFSW for several minutes (~2–4 min) to achieve a steady‐state signal. At the end of each measurement, 800 U L^−1^ SOD (Sigma) was added to seawater samples. A small fraction of the superoxide signal is a result of reagent autooxidation; thus, this artifact is removed by taking the difference between two signals as follows. First, the chemiluminescent response, *R*, in the UFSW, FFSW, and AFSW was quantified relative to the SOD baseline and converted to concentration using the calibration sensitivity, *S*_*calibration*_ (Equation 1). Next, the total light‐independent superoxide concentration was determined using the difference between the UFSW signal and AFSW signal (Equation 2, Roe et al., 2016). The particle‐associated concentration was defined as the difference between the UFSW signal and FFSW signal (Equation 3). The primary assumption here is that the sources of superoxide in the AFSW are negligible; otherwise, the concentrations are underestimates of the true steady‐state dark values.

(1) $${\left[O_{2}^{\cdot -}\right]}_{\left(\text{UFSW},\;\text{AFSW},\text{or FFSW}\right)}=\frac{R_{\text{sample}}-R_{\text{sample}+\mathrm{SOD}}}{S_{\text{calibration}}}$$

(2) $${\left[O_{2}^{\cdot -}\right]}_{\text{total}}={\left[O_{2}^{\cdot -}\right]}_{\text{UFSW}}-{\left[O_{2}^{\cdot -}\right]}_{\text{AFSW}}$$

(3) $${\left[O_{2}^{\cdot -}\right]}_{\text{particle associated}}={\left[O_{2}^{\cdot -}\right]}_{\text{UFSW}}-{\left[O_{2}^{\cdot -}\right]}_{\text{FFSW}}$$

Calibrations were conducted using potassium dioxide (Sigma) as detailed previously (Zhang et al., 2016). Briefly, a primary stock solution containing potassium dioxide was prepared and quantified spectrophotometrically (Abs_240_). To prepare the calibration standards, the primary stock solution was further diluted with the calibration matrix to a final superoxide concentration of 5–41 nM. Both primary stock solution and calibration standards were prepared immediately before the analysis. The corresponding chemiluminescent signals were recorded and extrapolated back to the time when the primary standard was quantified, using first‐order decay kinetics. The half‐life of superoxide in AFSW ranged from 0.26 to 0.49 min, and the extrapolation time was 0.5–1 min. Calibration curves were constructed based on the linear regression of the natural logarithm extrapolated chemiluminescent signals versus superoxide concentrations in the calibration standards. Calibrations yielded highly linear curves (e.g., *R* ^2^ > 0.9), with a sensitivity, *S*_*calibration*_, of 0.16 ± 0.04 (average and standard deviation of different water depths) counts per pM.

*Decay and production rates*. At a subset of stations and depths, superoxide decay rates within unfiltered waters were quantified. Decay rate constants of superoxide were determined by spiking in known concentrations of a calibrated potassium dioxide stock and measuring superoxide decay over time as discussed above. The decay constants were obtained by modeling data using a pseudo first‐order decay equation (Armoza‐Zvuloni & Shaked, 2014; Shaked & Armoza‐Zvuloni, 2013). Waters were spiked with superoxide levels ~2–3 times measured in situ concentrations. Production rates were calculated using the measured steady‐state superoxide concentrations and modeled decay rate constants for each water sample (Roe et al., 2016).

We note that hydrogen peroxide measurements were also collected during this study using the Amplex Red fluorescence assay as previously described (Rose et al., 2010). We found that hydrogen peroxide concentrations in the aphotic zone were consistently 20–50 nM higher than values previously reported (Yuan & Shiller, 2001). While it is possible that these are true concentrations, it is also possible that some yet uncharacterized component of the seawater, likely within the elevated coastal DOC reservoir, causes an interference with the fluorescence assay. Such interferences have been identified with other organic compounds (Serrano et al., 2009). In any case, these concentrations require further validation. We have no reason to suspect that these higher than expected hydrogen peroxide concentrations were the result of contamination considering the trace metal clean protocols used to collect and process samples and given the results of trace metal analyses, including Mn and Hg, that were conducted on the very same samples (e.g., Oldham et al., 2020).

### 2.3 Chlorophyll

In the dark, 250 ml of seawater was filtered onto 25 mm GF/F filters. Samples were stored in the dark at −80°C until analyzed according to protocols adapted from Strickland and Parsons (Strickland & Parsons, 1972). Briefly, samples were extracted in 90% acetone in the dark (4°C, 9 hr) and measured using a 10 AU fluorometer (Turner). Sample signals were calibrated using a chlorophyll‐a standard (Sigma C6144) and were corrected for pheopigments by accounting for the fluorescence of extracts before and after acidification in 0.003 M HCl.

### 2.4 Flow Cytometry

Flow cytometry samples were filtered (40 μm), preserved in 0.5% glutaraldehyde (final concentration), flash frozen in liquid nitrogen, and stored at −80°C prior to analysis. Counts (cells per milliliter) were obtained by pipetting 200 μl triplicate samples and filtered seawater blanks (0.2 μm) into 96 well plates and analyzing at a low flow rate (0.24 μl s^−1^) on a Guava® easyCyte flow cytometer (Millipore Sigma, Merck KGaA, Dermstadt, Germany). Three phytoplankton groups were distinguishable based on plots of red fluorescence and forward scatter (picoeukaryotes, nanoeukaryotes) and orange fluorescence and forward scatter (phycoerythrin‐containing *Synechococcus* spp.). Bacteria samples were diluted as needed with filtered (0.2 μm) seawater, stained with SYBR Green I (Invitrogen) according to the manufacturer's instructions, and incubated in the dark at room temperature for at least 30 min before analyzing on the Guava® easyCyte flow cytometer. Bacteria were distinguishable based on green and red fluorescence and green fluorescence and forward scatter. Analyses were performed on the Guava InCyte™ 3.1 software. Detection limits were calculated as 3 times the standard deviation of the filtered (0.2 μm) seawater blank.

### 2.5 DOC

Filtered water samples for total DOC were pipetted into acid‐washed combusted glass vials, acidified to pH = 2 with 12 M hydrochloric acid, and stored at 4°C until analysis on a Shimadzu TOC‐5050A total organic carbon analyzer. The coefficient of variability between replicate injections was less than 1%.

### 2.6 Nitrogen Speciation

Concentrations of nitrate plus nitrite were measured by chemiluminescence after reduction in a hot acidic vanadyl sulfate solution on a NOx analyzer (Braman & Hendrix, 1989). Concentrations of nitrite were quantified by using the Griess‐Ilosvay method followed by measuring absorption at 543 nm (Grasshoff et al., 1999), and nitrate was quantified by difference. Concentrations of ammonium were measured by fluorescence using the o‐phthalaldehyde (OPA) method (Holmes et al., 1999).

### 2.7 Data Analysis

Measurements collected in this study were analyzed using a combination of principal component analysis (PCA) and pairwise linear least squares regressions. All reported *p* values are produced from a two‐sample *t* test for equal means. PCA was performed with MATLAB and included 19 observations of parameters with continuous distributions (including superoxide measurements, CTD measurements, flow cytometry, and water chemistry) at 16 depths at which all observations were collected and include sample depth, temperature, chlorophyll concentration, PAR, beam transmission, salinity, dissolved oxygen concentration, nitrite concentration, ammonium concentration, nanoeukaryote concentration, picoeukaryote concentration, *Synechococcus* concentration, bacteria concentration, DOC concentration, superoxide concentration, AFSW decay rate constant, UFSW decay rate constant, fraction of steady‐state superoxide concentration from particles, and superoxide production rate.

## 3 Results

### 3.1 Site Biogeochemistry

**Figure 2:**
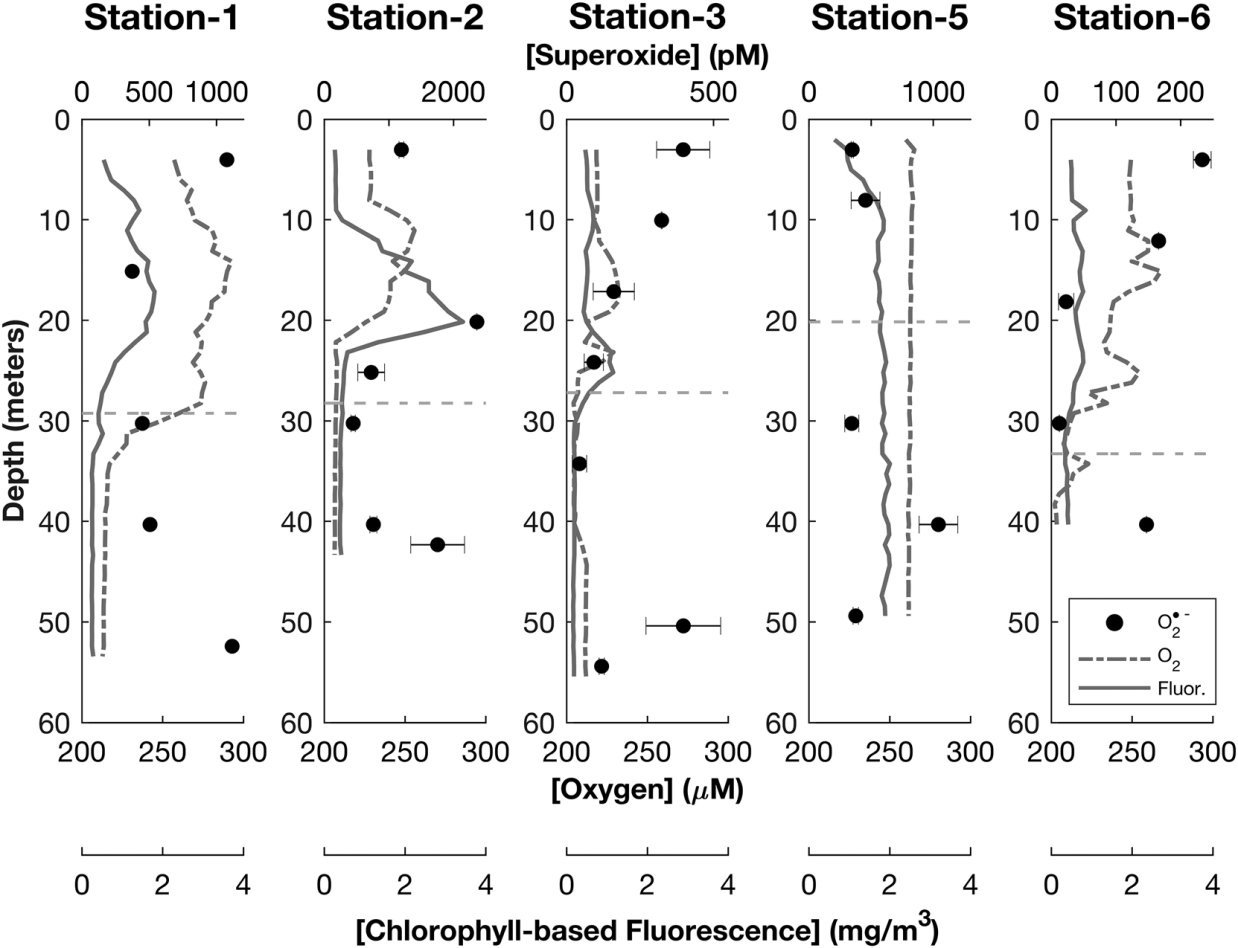
Water column profiles (on‐shelf locations) of light‐independent superoxide (pM; upper axis with black filled dots). Also plotted are the dissolved oxygen concentrations (𝜇M; lower primary axis with gray dashed line) and chlorophyll‐based fluorescence (mg m^−3^; lower secondary axis with solid gray line). The error bars represent the standard deviation between two replicates. The dashed horizontal gray line represents the 1% light level defined as the base of the photic zone.

**Figure 3:**
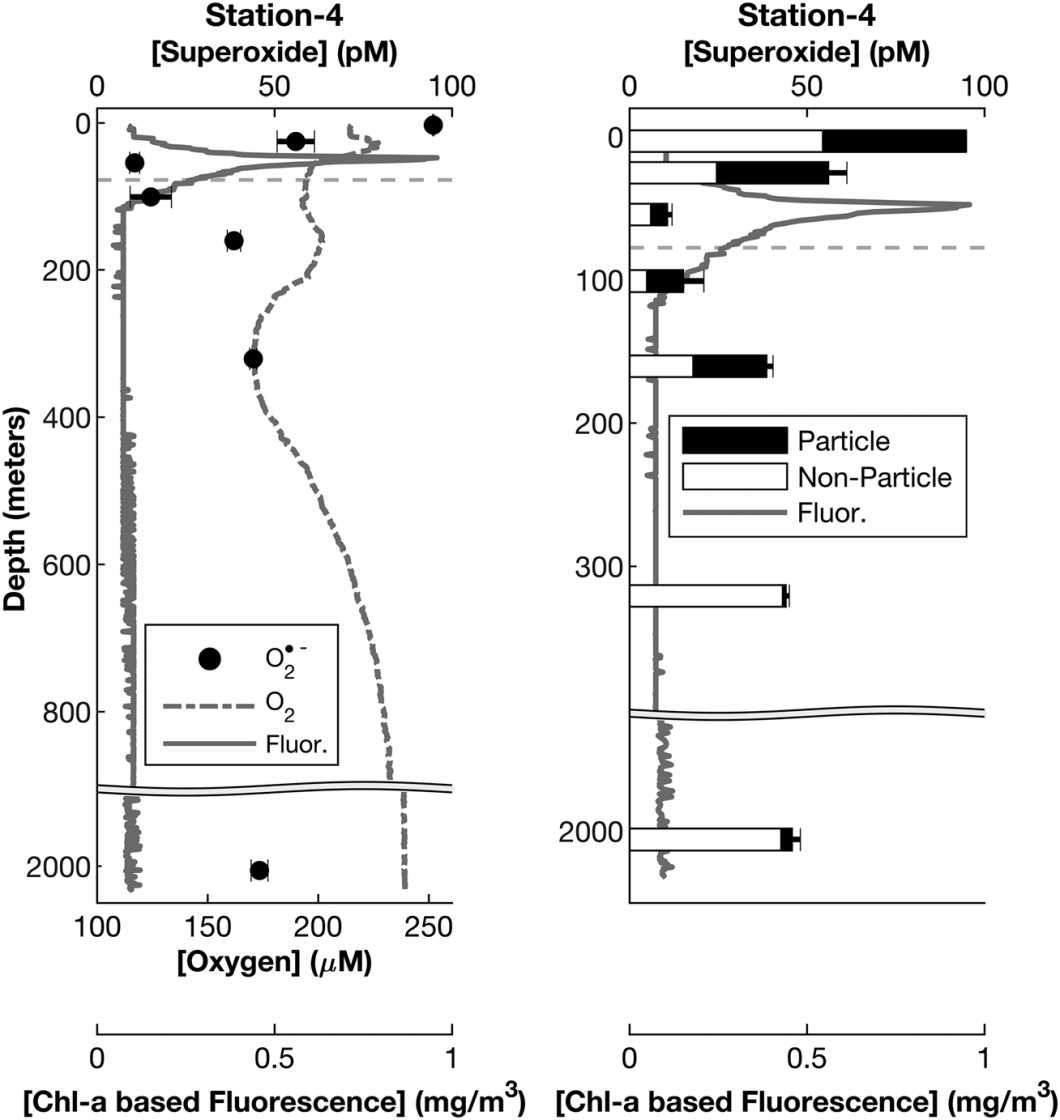
Water column light‐independent, steady‐state superoxide (pM; upper axis with black filled dots) concentrations at Station 4 (left). The fractional contribution of particle‐associated superoxide production to the total superoxide concentration is also shown (particle associated in black, nonparticle associated in white, right image). Dissolved oxygen concentration (𝜇M; lower primary axis with gray dashed line, left only) and chlorophyll‐based fluorescence (mg m^−3^; lower secondary axis with solid gray line) are also shown. The error bars represent the standard deviation between two measurements. The dashed horizontal gray line represents the 1% light level defined as the base of the photic zone. Note the *y* axis breaks at 900 m (left) and 450 m (right).

The five shelf stations and one off‐shelf station offered a range of hydrographic conditions with which to interrogate superoxide in the marine water column. Stations 1 (near Rhode Island) and 2 (near New Jersey) exhibited relatively distinct dissolved oxygen and chlorophyll a‐based fluorescence maxima between 10 and 25 m depth, with Station 2 having slightly higher fluorescence at the peak (Figure 2). Station 3 (near Maryland) had more understated dissolved oxygen and fluorescence maxima, at approximately 18 and 25 m, respectively. Station 6 (near New York) lacked a pronounced fluorescence maximum but showed multiple small peaks in dissolved oxygen throughout the photic zone. Temperature and salinity profiles at Stations 1, 2, 3, and 6 revealed a relatively shallow mixed layer depth, which was typically around 10 m depth (Figure S1). Station 5 (Georges Bank) demonstrated near‐uniform fluorescence, dissolved oxygen concentration, temperature, and salinity throughout the water column, indicating that the location was vertically well mixed (Figures 2 and S1). Station 4 (off‐shelf) had a mixed layer depth of approximately 10 m, a dissolved oxygen maximum at approximately 30 m, and a fluorescence maximum at approximately 46 m. The base of the photic zone at Station 4 is at approximately 77 m below the surface (Figures 3 and S2). There is also a secondary oxygen maximum below the photic zone (~170 m). PAR at all stations exhibited a smooth exponential decrease with increasing depth, with a peak irradiance ranging from ~1,000–2000 𝜇mol photons m^−2^ s^−1^ (Figures S1 and S2).

Flow cytometry measurements (including bacteria and group‐specific phytoplankton: picoeukaryote and nanoeukaryote and *Synechococcus*) demonstrated that all microbial populations reached a maximum concentration in the top 1–3 sample depths (~3 to ~15 m) and generally decreased with depth. An exception to this was the vertically mixed water column at Georges Bank that showed less structure in vertical profiles (Figures S4–S9 and Table S4). DOC ranged from 1–1.5 mg L^−1^ in the surface and decreased with depth (Figures S4–S9 and Table S2). Microbial cell counts and DOC in the shelf stations were all higher than comparable depths at the off‐shelf site.

### 3.2 Superoxide Concentrations

In the absence of light, steady‐state superoxide concentrations within unfiltered waters ranged from approximately 10 pM to as high as 2,400 pM (Figures 2 and 3, Table 1). The average light‐independent total superoxide concentration across all on‐shelf sample stations (570 ± 550 pM, *n* = 29) was significantly different from the average concentration at the off‐shelf station (44 ± 28 pM, *n* = 7, *p* = 0.017).

**Table 1:** Summary of Superoxide Measurements

Station	Depth (m)	Superoxide concentration (pM)^a^	Fraction of steady state superoxide concentration due to particles (%)	Superoxide decay rate constant in UFSW (s^−1^)	Superoxide decay rate constant in AFSW (s^−1^)
On‐shelf stations					
1	4.0	1,080 ± 10	—	—	0.020
	15.1	373 ± 3	—	—	0.017
	30.2	449 ± 2	—	—	0.016
	40.3	510 ± 10	—	—	0.018
	50.4	1,217 ± 2	—	—	0.018
	52.4	1,120	—	—	—
2	3.0	1,190 ± 40	31.4	—	0.018
	20.2	2,360	100	—	0.017
	25.2	700 ± 200	66.3	—	0.016
	30.2	450 ± 30	100	—	0.016
	40.3	760 ± 50	57.4	—	0.015
	42.3	1,800 ± 400	42.0	—	0.017
3	3.0	400 ± 90	100	0.026	0.015
	10.1	323 ± 1	92.7	0.030	0.014
	17.1	160 ± 70	100	0.047	0.014
	24.2	90 ± 30	100	0.058	0.019
	34.3	40 ± 20	100	0.037	0.015
	50.4	400 ± 100	100	—	0.016
	54.4	120 ± 10	100	0.047	0.019
5	3.0	350 ± 10	100	0.091	0.037
	8.1	500 ± 100	81.5	0.125	0.020
	30.2	340 ± 60	79.5	0.065	0.018
	40.3	1,000 ± 200	70.3	0.031	0.016
	49.4	380 ± 20	80.5	—	0.024
6	4.0	230 ± 10	100	0.051	0.020
	12.1	170 ± 20	91.1	—	0.010
	18.1	20 ± 10	44.8	—	0.031
	30.2	12 ± 2	88.8	—	0.012
	40.3	150	100	0.031	0.013
Off‐shelf stations					
4	3.0	95 ± 1	42.5	—	0.006
	25.2	56 ± 5	56.1	0.039	0.001
	54.4	11 ± 1	43.1	0.026	0.005
	100.8	15 ± 6	67.5	0.021	0.007
	160.3	39 ± 2	53.5	0.024	0.007
	320.7	44 ± 1	2.0	0.058	0.006
	2,005.8	46 ± 2	6.6	0.026	0.005

a Values reported as mean and standard deviation of replicates (*n* = 2 for superoxide). Omission of standard deviation indicates measurement of single replicate.

At some Stations, such as 1 and 6, light‐independent, steady‐state superoxide concentration profiles were C shaped, with elevated concentrations at the surface and deepest sample and lower concentrations in‐between (Figure 2). At Station 2, elevated superoxide concentrations were observed near the surface, the fluorescence maximum, and lowest sample depth. At Station 3, we observed a steady decline in superoxide concentration from the surface to below the photic zone but observed elevated concentrations close to the seafloor (3 m above seafloor). Station 5 steady‐state superoxide concentrations were largely invariant and centered around 400 pM, with the exception of one sample at 40 m depth with a significantly elevated concentration. At the off‐shelf site (Station 4), the 7‐point depth profile exhibited a maximum closest to the surface (~100 pM) and a minimum near the fluorescence maximum (~10 pM) that rebounded to near ~40 pM for the remaining depths (Figure 3).

### 3.3 Particle‐Associated Superoxide

**Figure 4:**
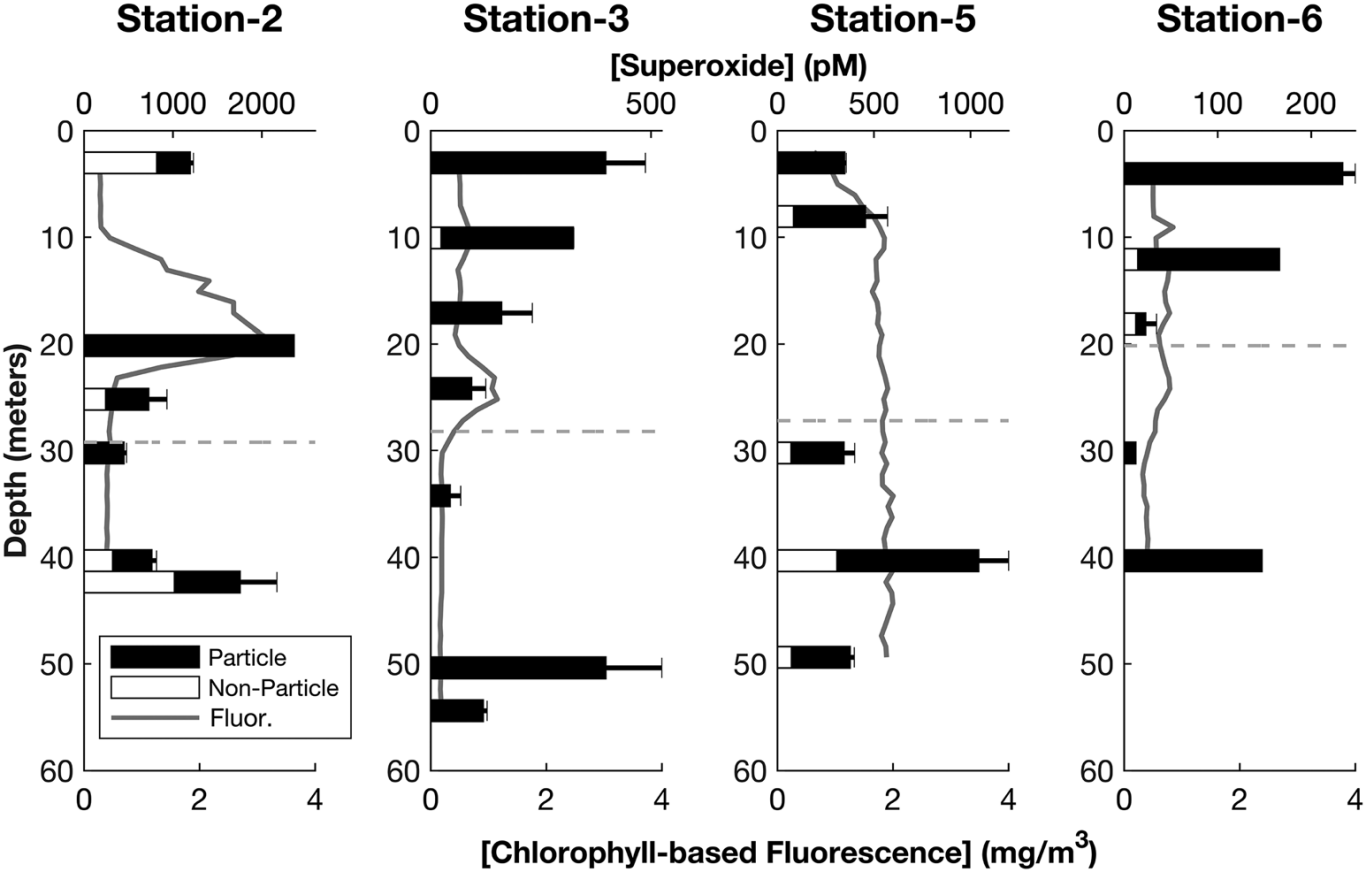
Water column profiles of four on‐shelf sampling locations where particle‐ and nonparticle‐associated superoxide concentrations were measured. The total light‐independent superoxide concentrations (pM) are plotted as bar graphs with particle‐associated (black) and nonparticle‐associated (white) contributions shown. Water column chlorophyll‐based fluorescence (mg m^3^) is plotted in gray. The error bars represent the standard deviation between two replicates for total light‐independent superoxide concentration.

Across all sample locations and depths, the light‐independent, steady‐state superoxide concentration was lowered by filtration (Figures 3 and 4 and Table 1), such that an average of 73% of superoxide can be attributed to particles (calculated as filtered signal divided by unfiltered superoxide signal). However, there was significant variability across sample location and depth, with particles accounting for as little as 2% and as much as 100% of the total superoxide concentration. The fractional contribution of particles to the superoxide concentration in on‐shelf sample locations was 84 ± 22% (*n* = 23), which was significantly different from that of the off‐shelf sample location (39 ± 25%, n = 7, *p* = 0.015).

In the four shelf stations in which particle association of superoxide was investigated (Stations 2, 3, 5, and 6), there was no single trend that dominated. At Station 2, superoxide was primarily particle associated at the fluorescence maximum and at the base of the photic zone, but nonparticle‐associated superoxide production contributed significantly to the steady‐state superoxide concentration at the remaining four depths (Figure 4). At all depths at Station 3, a majority of superoxide was particle associated. At Station 5, superoxide in the shallowest depth was all particle associated and approximately 70–80% particle associated at all other depths. At Station 6, particles accounted for 100% of superoxide production at 4 and 40 m and accounted for as little as half at intermediate depths. In the deep, off‐shelf station, steady‐state superoxide concentrations were of mixed origin (particulate and dissolved) at all depths, with particles responsible for approximately 40–70% of superoxide production in the top five sampling depths (down to 160 m) (Figure 3). Particulate superoxide production contributed very little in the deepest depths of Station 4, comprising 2% and 7% at 320 and 2,005 m, respectively.

### 3.4 Decay Rates

Superoxide pseudo first‐order decay rate constants ranged from 0.021 to 0.125 s^−1^ for UFSW and 0.001 to 0.037 s^−1^ for aged filtered seawater (Table 1). Superoxide decay rate constants were significantly different between aged filtered and unfiltered samples (two sample *t* test, *p* < 0.001). Decay rate constants of superoxide in UFSW, where measured (Stations 3–6), were typically the highest at intermediate depths. An exception to this was seen in Station 4 where the highest decay rate constant occurred at 320 m. Decay rate constants in AFSW exhibited a much smaller dynamic range than those seen in UFSW. At Stations 1, 2, and 5, the highest AFSW decay rate constant occurred in the shallowest depth, while the most rapid decay was seen in intermediate depths elsewhere. Average AFSW decay rate constants in the off‐shelf station were approximately fourfold lower than those observed on the shelf.

### 3.5 Superoxide‐Biogeochemistry PCA

The underlying relationships between the set of observations collected in this study were investigated further using PCA. PCA results are shown in the supporting information (Figure S3). In brief, the principal components explained the following fractional variance: PC1: 52.0%, PC2: 15.3%, PC3: 13.3%, and PC4: 5.5%, and all remaining PCs (5–15) explain less than 5% each of the total variance.

**Figure 5:**
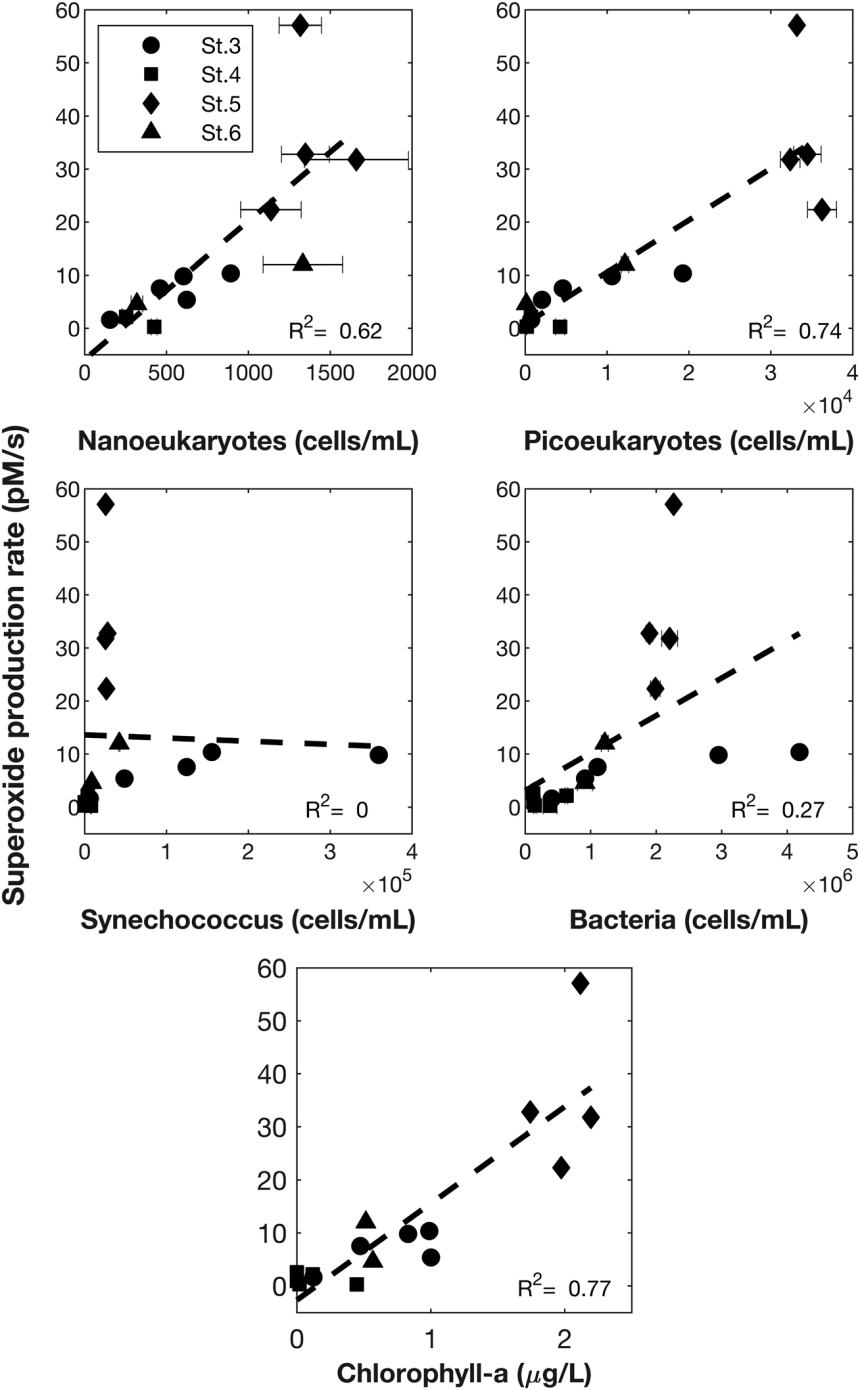
Linear least squares fits for superoxide production rate as a function of nanoeukaryotes (top left), picoeukaryotes (top right), *Synechococcus* (middle left), bacteria (middle right), and chlorophyll concentration (bottom). Superoxide production rates were determined at all locations where superoxide decay rate constants were determined in unfiltered seawater. Error bars on cell counts represent one standard deviation of triplicate analysis.

PC1 and PC2, which together explain 67.3% of the observed variance, show a clustering in Quadrant III (negative PC1, negative PC2) of light‐independent, steady‐state superoxide concentration and superoxide production rate along with several other observations including eukaryote abundance (both picoeukaryote and nanoeukaryote), chlorophyll, dissolved oxygen, superoxide production rate, and both first‐order superoxide decay rate constants (Figure S3). The pairwise linear correlation between light‐independent, steady‐state superoxide concentration and these observations was all quite weak (all *R* ^2^ < 0.1), with the exception of superoxide production rate (*R* ^2^ = 0.51, Table S5), which was calculated using the steady‐state superoxide concentration. However, the pairwise correlation between superoxide production rate (where determined) revealed moderate correlations with chlorophyll concentration, picoeukaryote abundance, and nanoeukaryote abundance (*R* ^2^ = 0.6–0.8, Figure 5). Bacteria, *Synechococcus*, and total cell abundance (which is approximately equal to total bacteria abundance) did not correlate well with superoxide production rate (Figure 5).

## 4 Discussion

### 4.1 ROS Concentrations and Distribution

Light‐independent, steady‐state superoxide concentrations along the North Atlantic continental shelf exhibited a range of over 3 orders of magnitude, with the highest values (in excess of 1 nM) observed in waters collected both in and below the photic zone (Table 1, Figures 2 and 3). These values are generally in good agreement with previous measurements of light‐independent superoxide in productive ocean water, which include the Costa Rica dome and Gulf of Alaska (10 to >500 pM) (Hansard et al., 2010; Rose, 2012; Rose et al., 2008b). Given that the chosen sample locations and time of year were meant to interrogate ROS concentrations within productive marine surface waters, these values are likely near the high end of microbially mediated, light‐independent superoxide levels in the marine water column. Although these superoxide concentrations are among the highest water column values reported, we note that they are still ~2 orders of magnitude lower than values reported for sunlit coral reef ecosystems (Diaz et al., 2016; Grabb et al., 2019).

Differences between the physical and chemical environments in the shelf stations offer insight into some of the influences on superoxide in coastal seawater. Most of the shelf stations in this study had elevated light‐independent, steady‐state superoxide concentrations in shallow photic water and in aphotic water close to the seafloor (Figure 2). The clear exception to this is at Station 5, where the vertically mixed waters eliminated the physical and chemical gradients present in the other shelf stations (Figure S1). Superoxide concentrations at four of the five sampling depths at Station 5 fell within a relatively narrow range, mirroring the uniformity of the water column physical and chemical characteristics. The highest light‐independent, steady‐state superoxide concentration was observed in the near‐surface waters of Station 2 (~2,400 pM, Figure 2), coincident with the highest fluorescence readings (and corresponding chlorophyll concentrations) in these productive, near‐shore waters. While this anecdotal evidence does point to the likely contribution of living and/or organic seawater constituents to steady‐state superoxide concentration, none of these metrics (chlorophyll or DOC) were particularly robust indicators of steady‐state superoxide concentrations (*R* ^2^ = 0.06 and 0.01 respectively, Table S5). Chlorophyll does, however, demonstrate a stronger relationship to light‐independent superoxide production rate (*R* ^2^ = 0.77, Figure 5), a point that we explore in more detail in the next section.

Further insight into the environmental controls on superoxide production can be gained from contrasting the five on‐shelf sampling locations (Stations 1, 2, 3, 5, and 6) with the single off‐shelf sampling location (Station 4). Station 4 had lower chlorophyll and cell counts of all types than the shelf stations (Figures S4–S9). Surface light‐independent, steady‐state superoxide concentrations peaked at 95 pM in the off‐shelf station, as compared to >230–2,400 pM in the more productive on‐shelf waters (Figures 2 and 3). Light‐independent superoxide concentrations at Station 4 reached a minimum at the bottom of the photic zone, which is similar to what we observed at the other shelf stations. The superoxide concentration at Station 4 increased to ~40 pM at depths below 100 m. Thus, factors typically associated with productive coastal waters are associated with elevated superoxide concentrations, which are explored quantitatively below.

### 4.2 Controls on Superoxide Distributions

Since superoxide in the ocean has a wide range of potential sources and sinks that vary spatially and temporally, there appears to be no single biological, physical, or chemical influence that is a consistent, robust predictor of superoxide concentrations in seawater. There are, however, some factors that are closely related to superoxide production and steady‐state concentration.

For example, particles play a central role in extracellular superoxide production. In this study, particle‐associated production accounted for an average of 73% of the steady‐state superoxide concentration across all stations and depths. Marine particulate matter is a complex mixture of living and nonliving components that can include mineral (of both lithogenic and biogenic origin), whole cells, fecal pellets, and a wide range of adsorbed constituents (Goutx et al., 2007). The filter pore size, 0.2 μm, was chosen primarily to investigate the role of microbes in light‐independent ROS production. We make the assumption that particle‐associated superoxide production is primarily associated with microbes, but we recognize that other particle‐associated abiotic factors (e.g., mineral surfaces [Schoonen et al., 2006; Zent et al., 2008] and autooxidation of some functional moieties [Cross & Jones, 1991]) may be at play and should be the subject of future investigation. Microbes produce extracellular superoxide via membrane bound and transmembrane oxidoreductase enzymes. This avenue of extracellular ROS production has been documented across the domains of Bacteria and Eukarya, including phototrophs and heterotrophs (Diaz et al., 2013, 2018; Diaz & Plummer, 2018; Hansel et al., 2016; Plummer et al., 2019; Rose et al., 2008b; Sutherland et al., 2019). Within eukaryotic phytoplankton, the transmembrane NOX family NADPH‐oxidases have been implicated in extracellular ROS production (Anderson et al., 2016; Kustka et al., 2005; Saragosti et al., 2010). Although less well studied, transmembrane NOX have also been recently identified in bacteria (Hajjar et al., 2017). In addition, soluble extracellular ROS‐producing enzymes have been identified in marine diatoms and heterotrophic bacteria (Andeer et al., 2015; Diaz et al., 2019), but the diversity of superoxide‐producing enzymes is not well known and likely quite broad.

Superoxide decay also plays a critical role in regulating ROS in the environment. Pseudo first‐order decay rates of superoxide in UFSW samples, where measured, ranged from 0.021 to 0.125 s^−1^, with an average value of 0.048 ± 0.027 s^−1^ (Table 1). These rates are comparable in magnitude to previously reported decay rate constants of superoxide in marine waters (Heller & Croot, 2010b, 2010a). Filtering and aging the seawater samples overnight with DTPA (AFSW sample, i.e., removal of particles and complexation of dissolved metals) consistently yielded slower decay kinetics (range: 0.001 to 0.037 s^−1^, mean: 0.014 ± 0.007 s^−1^). On average, the aged, filtered sample had a decay rate constant that was 68% lower than the unfiltered sample. The primary sinks of superoxide in seawater include enzymatic elimination, reactions with dissolved metals, and reactions with dissolved constituents of seawater such as DOC (Wuttig et al., 2013a). In one previous study, an average of two thirds of superoxide decay was mediated by some combination of microbes and dissolved metals, with the remaining third resulting from dissolved seawater components, likely DOC (Wuttig et al., 2013a). The calculated superoxide production rates at steady state (product of first‐order decay rate constant and steady‐state concentration) ranged from ~1–200 nM hr^−1^ and were generally higher at on‐shelf sites than the off‐shelf site by approximately an order of magnitude. While decay rates generally decreased with depth, this was not always the case. It is clear that changes in both production and decay contribute to the dynamics of superoxide in the water column.

To further explore some of the underlying aspects of the seawater chemistry and microbial factors that might influence ROS production and decay, including those that may be active in particles, we turn to the results of the PCA which included 19 observations across 16 depths where all observations were made. A plot of components PC1 and PC2 (together explaining approximately 67% of the total variance, Figure S3) demonstrates that total light‐independent, steady‐state superoxide concentration, superoxide production rate, and superoxide decay rate constants cluster in the same quadrant (QIII, negative PC1, negative PC2) as chlorophyll, eukaryote abundance, and dissolved oxygen. We followed up each of these potential relationships with a pairwise linear correlation study of the depths at which all observations were made. Superoxide production rate exhibited a relatively strong relationship with chlorophyll (*R* ^2^ = 0.77), followed closely by abundances of picoeukaryotes (*R* ^2^ = 0.74) and nanoeukaryotes (*R* ^2^ = 0.62, *n* = 16, Figure 5). These correlations implicate eukaryotic phytoplankton as important contributors to particle‐associated ROS. This is consistent with previous studies that have demonstrated that eukaryotic phytoplankton produce extracellular superoxide at the highest rates of any widespread marine microbe and are known to produce extracellular superoxide in the dark (Diaz et al., 2019; Plummer et al., 2019; Rose et al., 2008b; Sutherland et al., 2019). Interestingly, *Synechococcus* abundance across all stations does not correlate to superoxide production rate (Figure 5), suggesting that eukaryotic phototrophs play a much more dominant role in ROS production in this coastal setting. These findings are largely consistent with those from a similar study in a coastal upwelling region in the Pacific, in which production rates were not correlated with *Synechococcus* abundance (Rose et al., 2008b). Although heterotrophic bacteria are known to be prolific producers of extracellular ROS (Diaz et al., 2013) and represent the largest microbial group in every sample location, the correlation between their abundance and superoxide production was poor (*R* ^2^ = 0.27). One factor that may underlie this poor correlation is that different bacterial groups may have wildly different production rates (Diaz et al., 2013). Another factor may be that microbial ROS production and degradation exhibit temporal variability in response to environmental factors (Morris et al., 2016). It is important to note that while these correlations are observed in the aggregate, similar tends are not observed within an individual station. One possible explanation for this effect may be competing influences of biogeochemical factors that vary laterally (i.e., distance from shore) with those that vary with depth in the water column.

Dissolved‐phase superoxide production accounted for the highest proportion of total superoxide in the deep aphotic water in the single off‐shelf station (Figure 3). Conversely, filtration consistently removed most light‐independent, steady‐state superoxide in the on‐shelf sampling locations (Figure 4). This difference suggests that seawater parameters that typically vary between productive surface waters and deep aphotic waters (e.g., nutrient/metal concentrations, cell type and abundance, and DOC) may underlie the higher proportion of dissolved‐phase light‐independent superoxide production in the off‐shelf station. We may be observing biologically mediated superoxide production that is the result of unattached extracellular enzymes. This has been documented in cell cultures but has not been explored in natural seawater previously (Andeer et al., 2015; Chaput et al., 2019; Diaz et al., 2018). In a similar vein, nonenzymatically mediated extracellular superoxide production may arise from the auto‐oxidation of organic matter. ROS, including hydrogen peroxide and hydroxyl radical, have been shown to result from dark reactions between molecular oxygen and dissolved organic compounds, indicating that superoxide may also form in this way (Page et al., 2012; X. Yuan et al., 2017). Microbial metabolites also produce ROS through auto‐oxidation, including thiols, flavins, quinones, catecholamines, and pterins (Cross & Jones, 1991).

Redox transformations of dissolved metals may also contribute to dissolved‐phase superoxide production (Halliwell & Foyer, 1976). Metals liberated from remineralized biomass or resuspended from underlying sediment may be oxidized by O_2_ via a single‐electron transfer, giving rise to superoxide. Some candidates would include common redox active metals in marine environments such as Fe, Mn, and/or Cu, which are typically present on the order ~1 nM in North Atlantic Deep Water. Such a reaction may also be reversible, producing a standing stock of superoxide that is actively shuttling electrons back and forth with dissolved metals and may not necessarily lead to significant net oxidation (e.g., Fe^2+^ oxidation by O_2_ followed by Fe^3+^ reduction by superoxide) (Wuttig et al., 2013a, 2013b). Lastly, it is important to recognize that the distinction between “dissolved” and “particulate” is a functional one based on filtration with 0.2 μm pore size filters. There is some fraction of microbes whose small size may allow them to pass through such a filter. For example, some rod‐shaped ammonia‐oxidizing archaea may have cell diameters as small as 0.15 μm (Santoro et al., 2015). While the data we collected in this study do not allow us to point toward one of these potential sources as the likely source of dissolved‐phase superoxide, these observations should be considered in the design of future experiments probing the nature of ROS in deep marine waters.

It is tempting to categorize several of the dissolved‐phase redox transformations discussed above as incidental or generally outside of the purview of microbial mediation. However, these radical reactions may be part of a larger chemical conversation between microbes and their environment. The production and consumption of ROS coupled with the redox cycling of labile redox‐active compounds in natural waters have been proposed as a mechanism by which microbial communities regulate the redox environment in and immediately surrounding the cell (Rose, 2016). While this notion is hypothetical, the broad utility of redox homeostasis could provide another explanation why the characteristic of extracellular superoxide production is so widespread (Diaz et al., 2013, 2019; Yuasa et al., 2020).

## 5 Summary and Conclusion

Over the last few decades, the paradigm of ROS production in the marine environment has evolved from a model that is primarily driven by photochemistry to one inclusive of photochemistry, abiotic reactions, and microbial production. Indeed, here we show that steady‐state, light‐independent superoxide concentrations in productive waters in the northeast coast of the United States range from 10 to >2,000 pM, with particles as the dominate source along the shelf. Our data also demonstrate the presence of dissolved‐phase superoxide production across a significant range of environmental conditions, most notably in deep waters off the continental shelf. Superoxide production rate is most closely associated with chlorophyll concentration and the abundance of picoeukaryotes and nanoeukaryotes, suggesting that eukaryotic phytoplankton are the main producers of light‐independent, particle‐associated superoxide within our study region.

The diversity of extracellular superoxide sources in the ocean has grown increasingly more complex as research over the last several decades has shown that superoxide production, and that of superoxide production in general, varies along dimensions of light, biological composition, and particle association. These biogeochemical pressures conspire to shape the steady‐state concentrations of ROS in the environment and in so doing shape the surrounding redox landscape and dictate the bioavailability of certain nutrients, the flow of electrons through biogeochemical systems, and selective pressures in a given environment.

## Supporting information

Supporting Information S1

## Data Availability

These data are publicly available through the Biological and Chemical Oceanography Data Management Office (BCO‐DMO) as data set version 2019‐04‐24 (https://doi.org/10.1575/1912/bco-dmo.765327.1).
