# Supplementary material for: Spatial Heterogeneity in Particle‐Associated, Light‐Independent Superoxide Production Within Productive Coastal Waters
Source: J Geophys Res Oceans. 2020 Oct 16;125(10):e2020JC016747. doi: 10.1029/2020JC016747 (PMC7685101; doi:10.1029/2020JC016747)
Supplement: Supplementary file 1 — Supporting Information S1 [file JGRC-125-e2020JC016747-s001.pdf]

JGR Oceans

Supporting Information for

Spatial Heterogeneity in Particle-Associated, Light-Independent Superoxide Production within Productive Coastal Waters

Kevin M. Sutherland[1,2,3], Kalina C. Grabb[1,2], Jennifer S. Karolewski[1,2], Sydney Plummer[4,5], Gabriela A. Farfan[6], Scott D. Wankel[1], Julia M. Diaz[4,5], Carl H. Lamborg[7], Colleen M. Hansel[1]

[1]Department of Marine Chemistry and Geochemistry, Woods Hole Oceanographic Institution, Woods Hole, MA 02543
[2]Department of Earth, Atmospheric and Planetary Science, Massachusetts Institute of Technology, Cambridge, MA 02139
[3]Current Address: Department of Earth and Planetary Science, Harvard University, Cambridge, MA 02138
[4]Skidaway Institute of Oceanography, Department of Marine Sciences, University of Georgia, 10 Ocean Science Circle, Savannah, GA 31411
[5]Current Address: Scripps Institution of Oceanography, University of California San Diego, La Jolla, CA 92093
[6]Department of Mineral Sciences, Smithsonian Institution, Washington, DC, 20560
[7]Ocean Sciences Department, University of California, Santa Cruz, Santa Cruz, CA 95064

Contents of this file

Figures S1 to S9
Tables S1 to S5

Temperature and Salinity Profiles of Sampling Stations

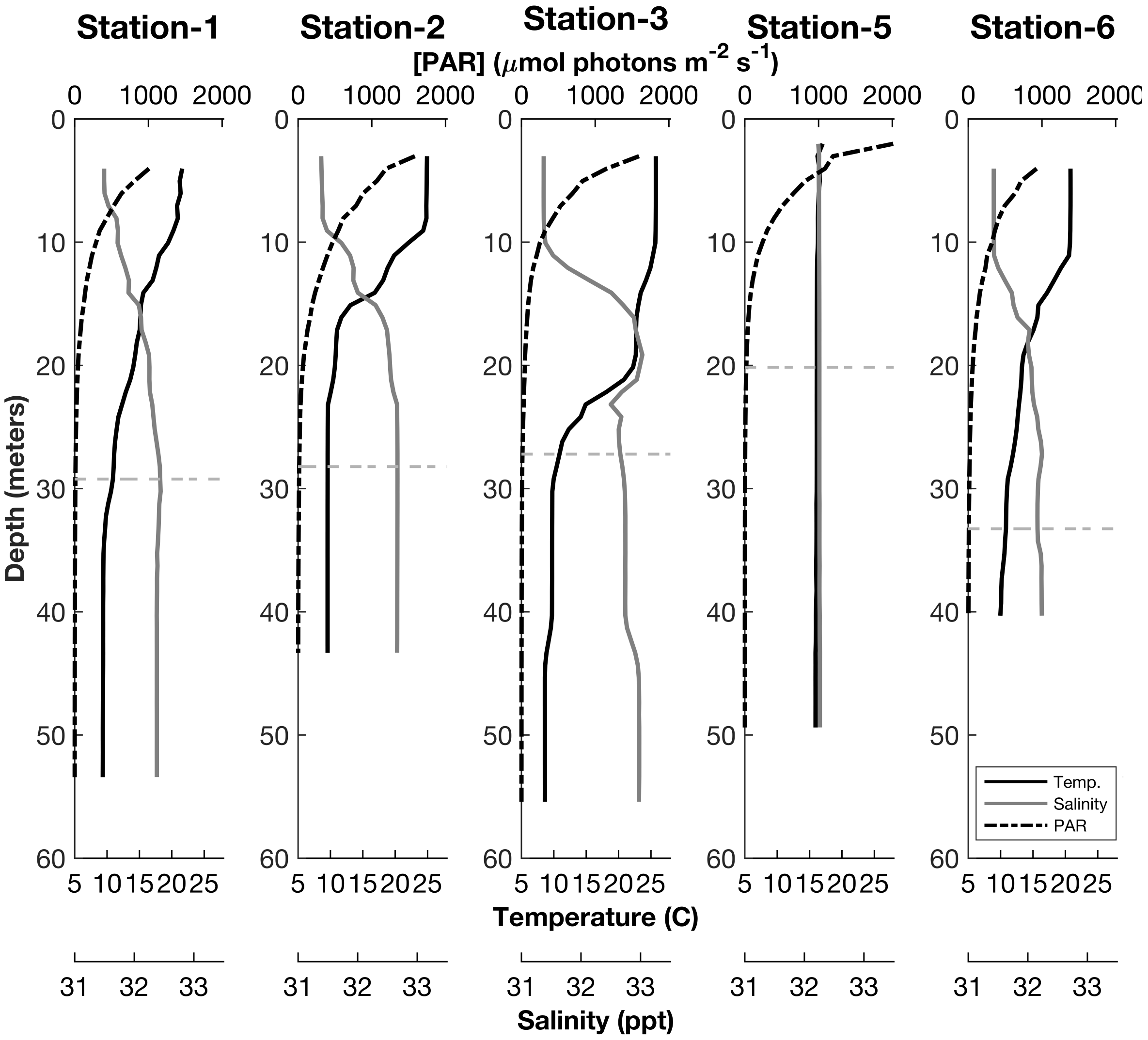

Figure S1- Temperature (black), salinity (gray), and PAR (black dot-dash) profiles of all shelf stations. Stations 1, 2, 3, and 6 demonstrate a relatively shallow mixed layer ranging from approximately 5 to 12 meters beneath the surface. Station 5 is vertically well-mixed. The base of the photic zone (defined here as 1% surface PAR) is shown with the horizontal dashed line.

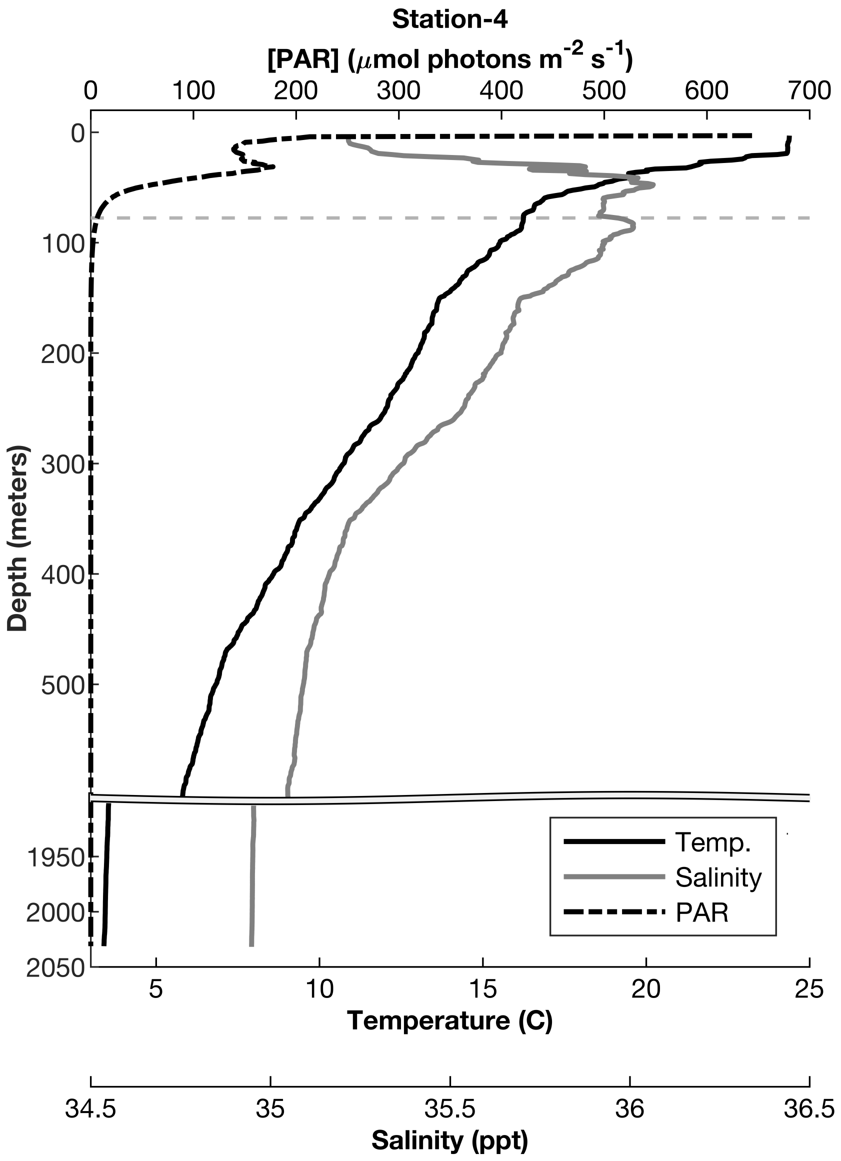

Figure S2- Temperature (black), salinity (gray), and PAR (black dot-dash) profile of Station 4. The base of the photic zone (defined here as 1% surface PAR) is shown with the horizontal dashed line.

Principal Component Analysis

We conducted a principal component analysis (PCA) on all observed variables for locations and depths where superoxide first-order decay rate constant was measured. This includes the following stations and depths (total of 17 depths): Station 3 (3.0 m, 10 m, 17.1 m, 24.2 m, 34.3 m, and 54.4 m), Station 4 (25.2 m, 54.4 m, 100.8 m, 160.3 m, and 320.7 m), Station 5 (3.0 m, 8.1 m, 30.2 m, 40.3 m, and 49.4 m), and Station 6 (4.0 m and 40.3 m). The observed variables that were included in the PCA were (total of 19 observed parameters): depth, temperature, chlorophyll, PAR, beam transmission, salinity, dissolved oxygen (O_2), nitrite (NO_2) concentration, ammonium (NH_4) concentration, nanoeukaryote (NanoEuk) abundance, picoeukaryote (PicoEuk) abundance, *Synechococcus* abundance, bacteria abundance, dissolved organic carbon (DOC) concentration, superoxide concentration, AFSW decay rate constant, percent particle contribution to superoxide concentration (particle contribution %), UFSW decay rate constant, and superoxide production rate.

The results of the PCA are plotted below in Figure S3A and Figure S3B. The fraction of the total variance explained by each principal component (PC) are: PC1: 52.0%, PC2: 15.3%, PC3: 13.3%, PC4: 5.5%, PC5: 4.6%, PC6: 3.3%, PC7: 1.9%, PC8: 1.5%, all remaining PCs (9-15) explain less than 1% of the total variance.

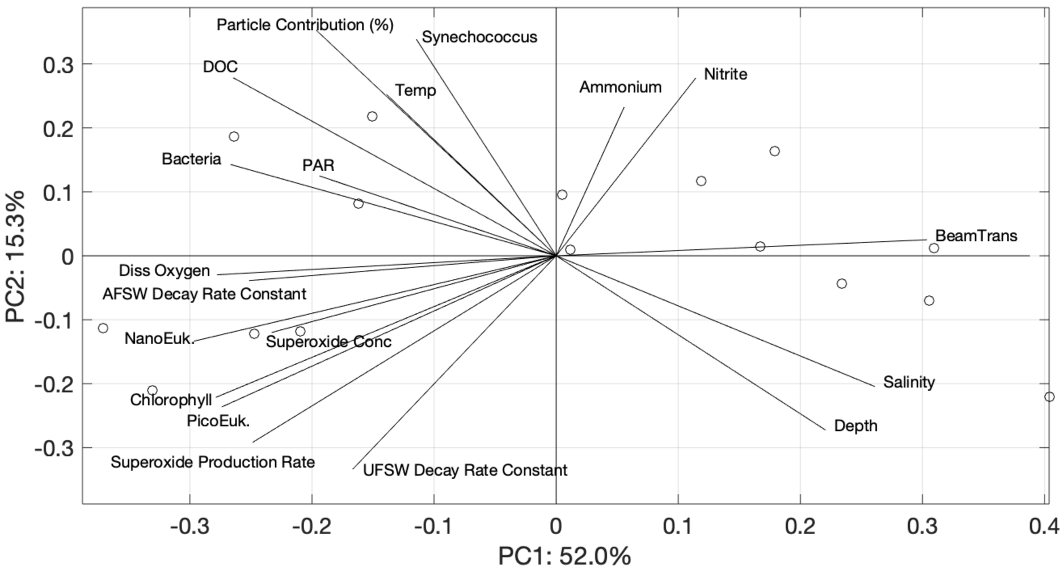

Figure S3A- Summary of PCA results plotted as PC1 vs. PC2.

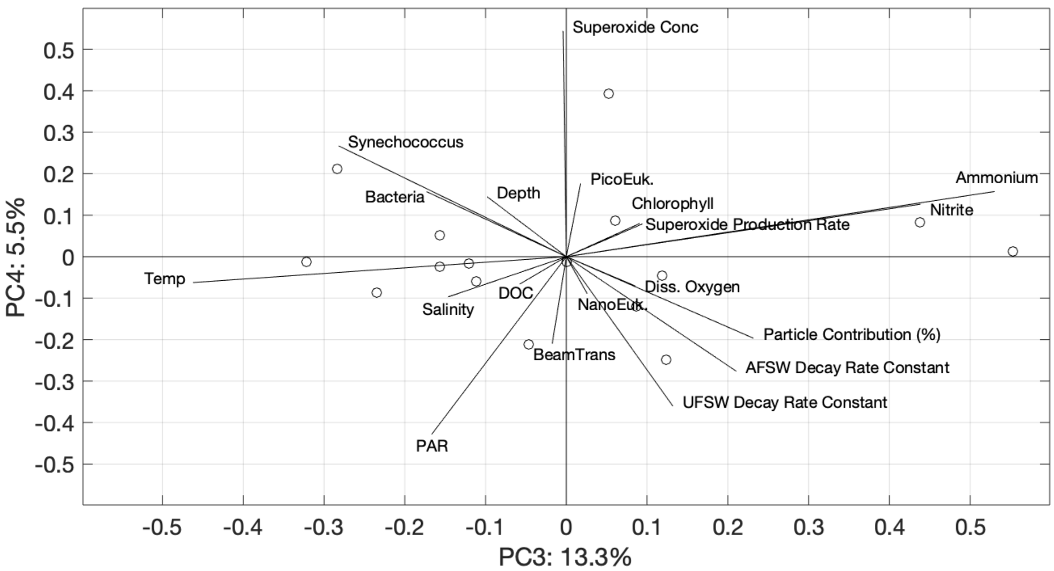

Figure S3B- Summary of PCA results plotted as PC3 vs. PC4.

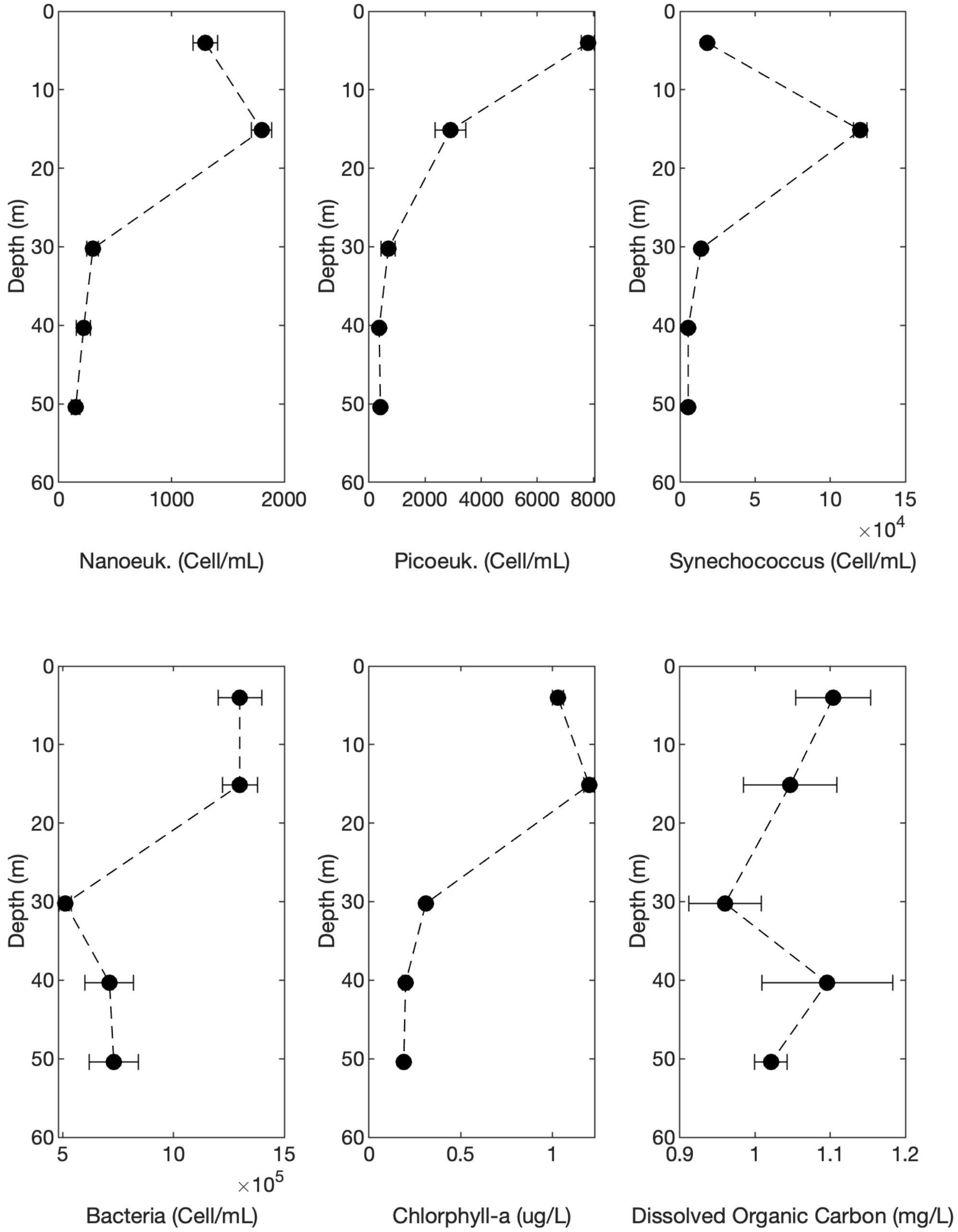

Figure S4- Summary of Station 1 flow cytometry, chlorophyll, and DOC measurements.

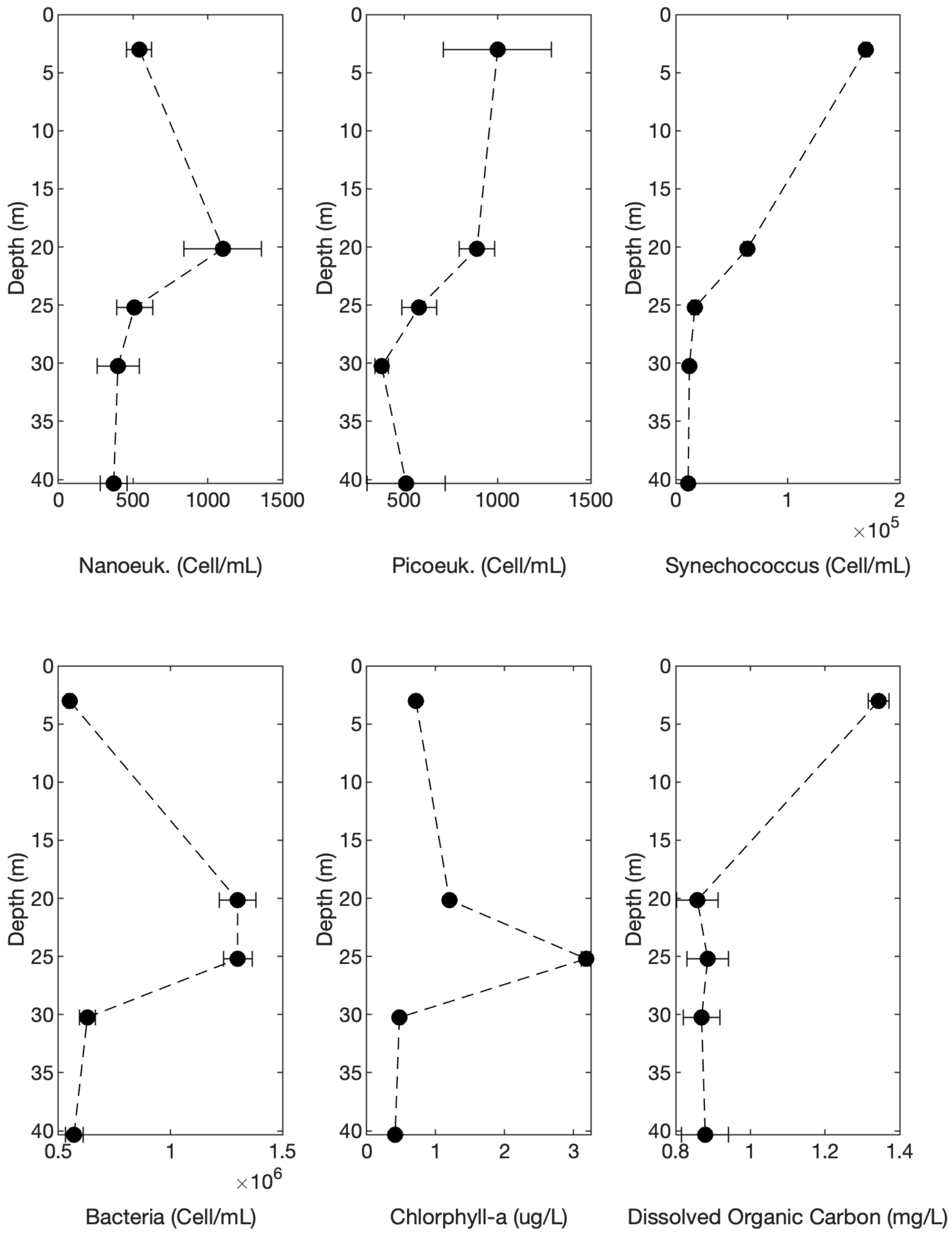

Figure S5- Summary of Station 2 flow cytometry, chlorophyll, and DOC measurements.

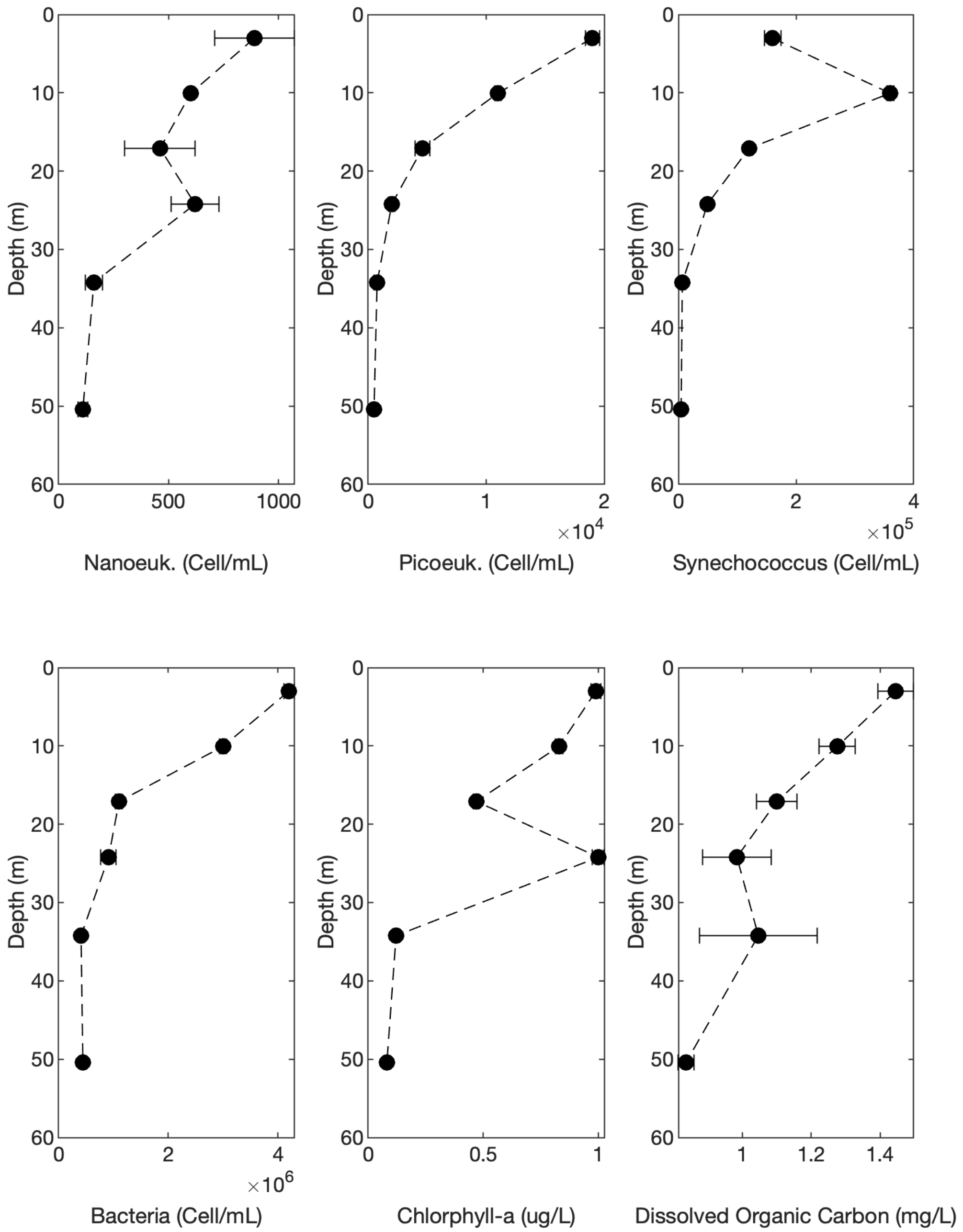

Figure S6- Summary of Station 3 flow cytometry, chlorophyll, and DOC measurements.

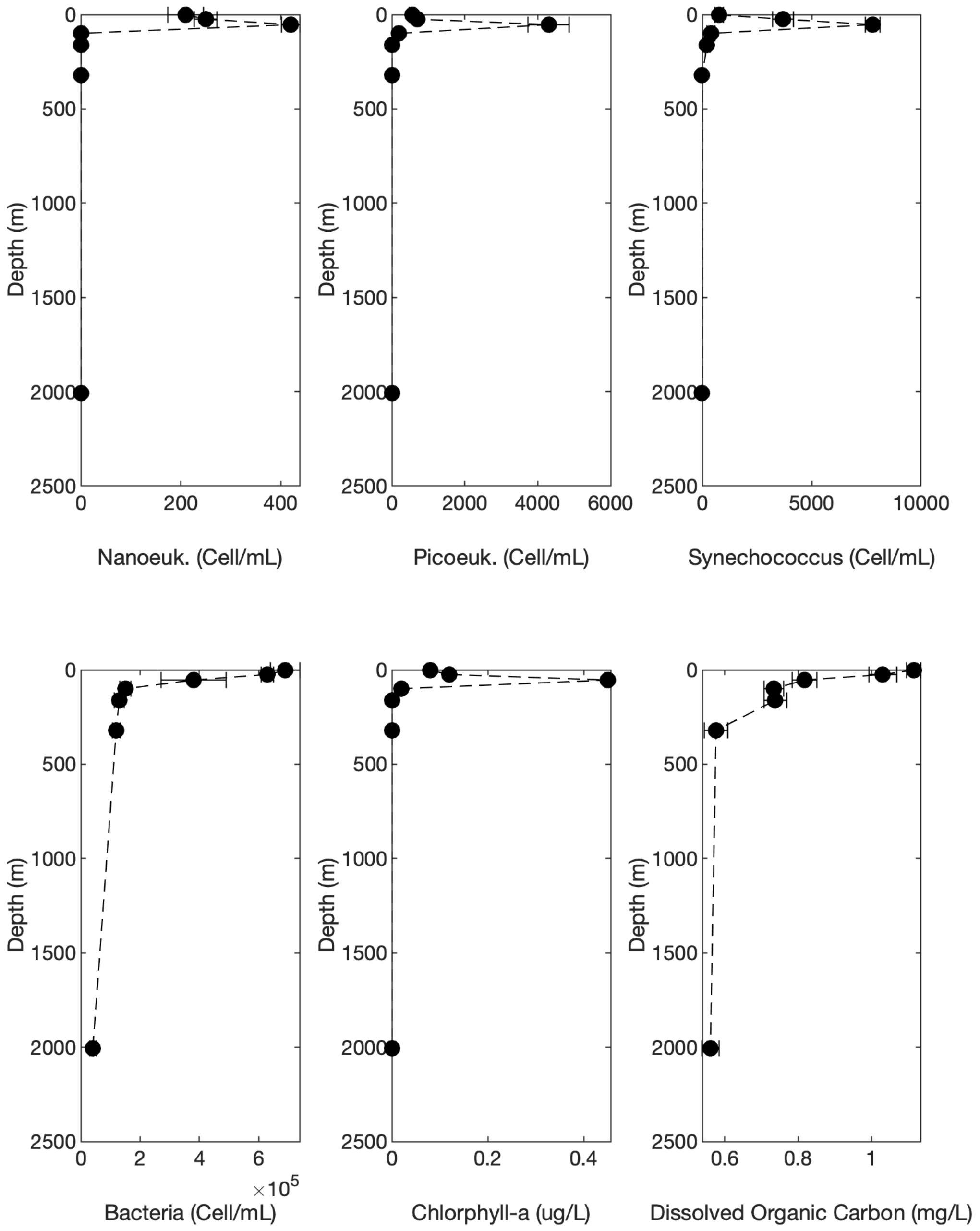

Figure S7- Summary of Station 4 flow cytometry, chlorophyll, and DOC measurements.

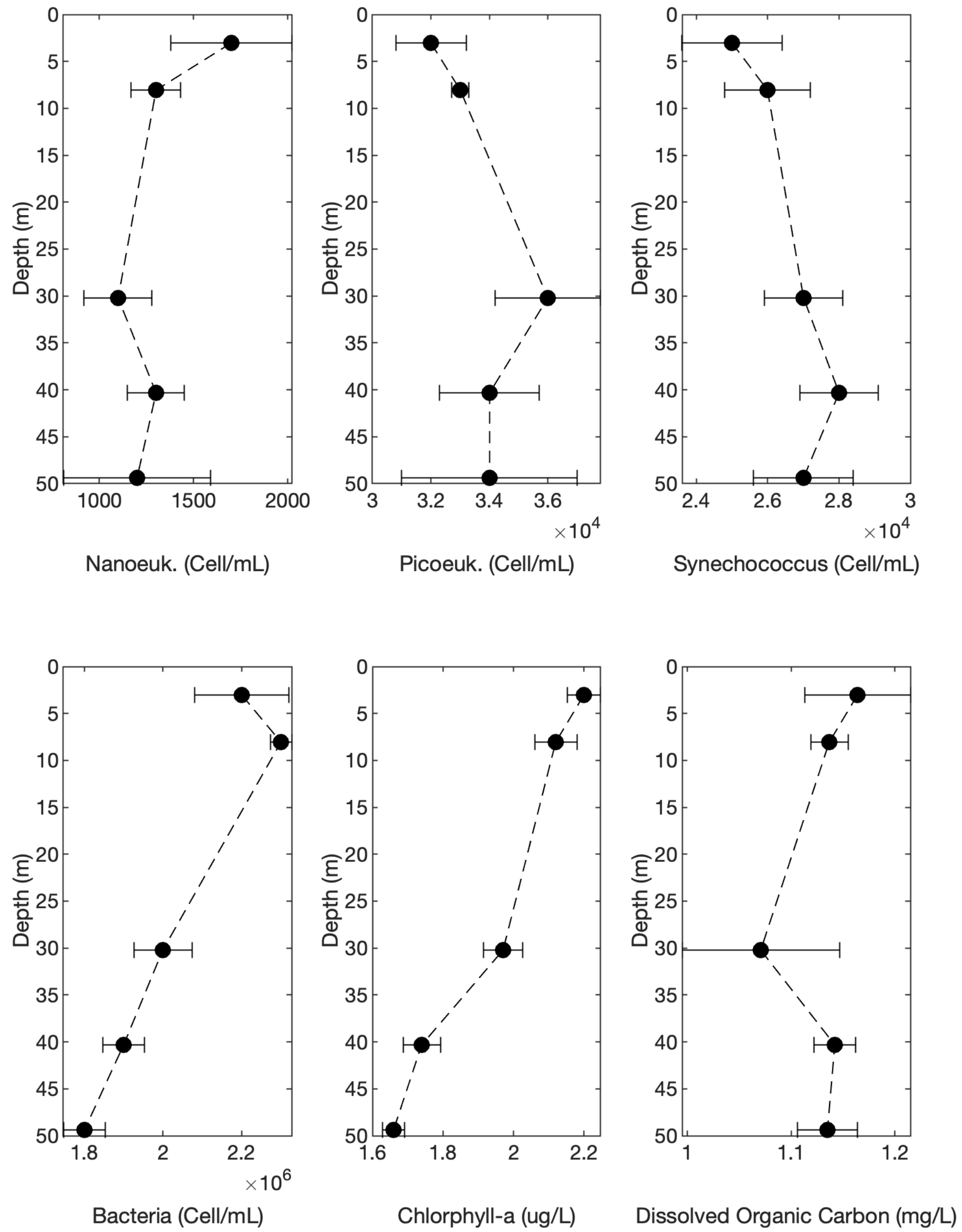

Figure S8- Summary of Station 5 flow cytometry, chlorophyll, and DOC measurements.

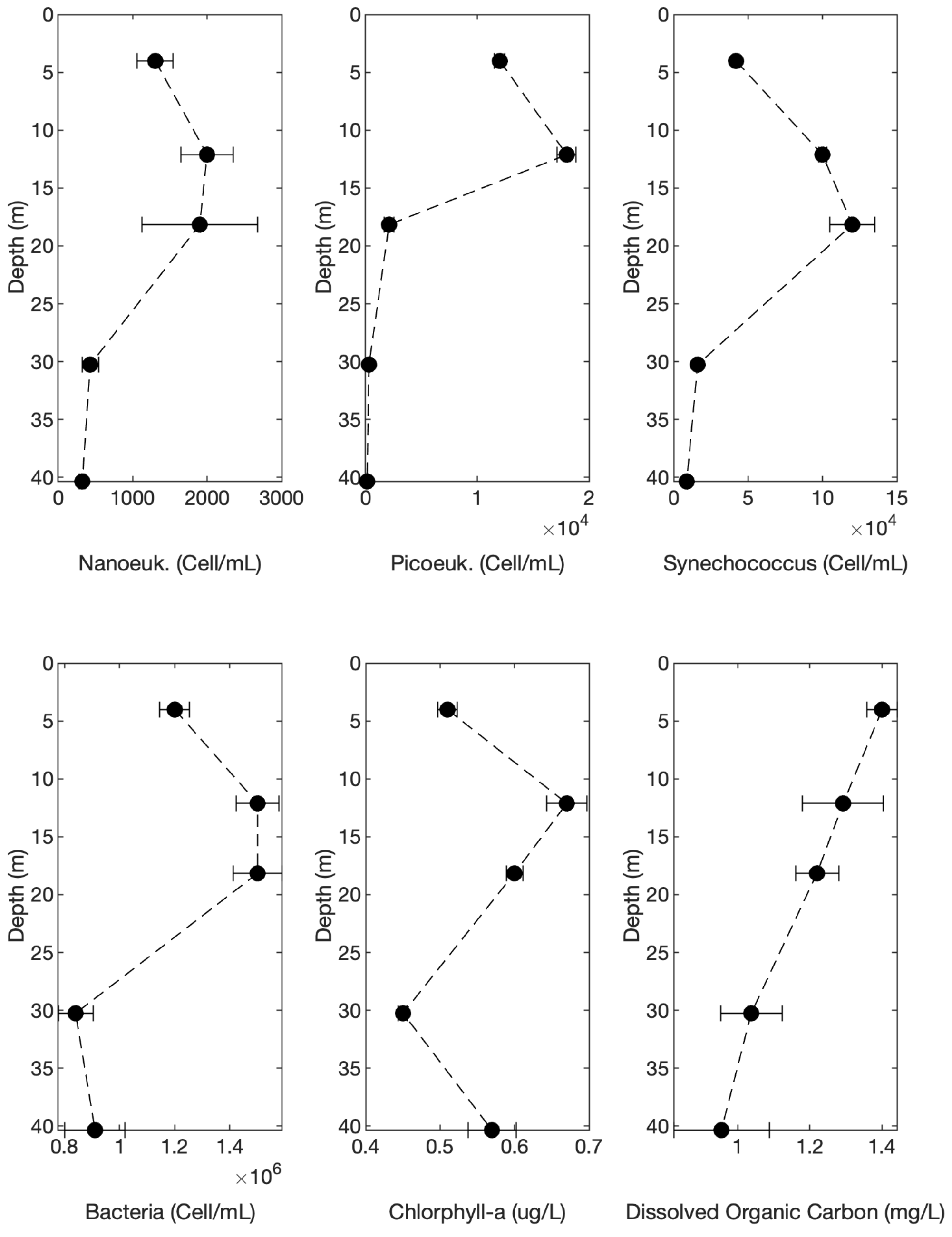

Figure S9- Summary of Station 6 flow cytometry, chlorophyll, and DOC measurements.

Table S1-Sampling Location, Date, and Time

Station		Lat	Long	Year	Month	Day	Deployment Time	Deployment Time
							hrs:min:sec in GMT	hrs:min:sec local time
1	Block Island	40.8722	-71.69217	2017	8	20	20:06:38	16:06:38
1		40.8722	-71.69217	2017	8	20	20:06:38	16:06:38
1		40.8722	-71.69217	2017	8	20	20:06:38	16:06:38
1		40.8722	-71.69217	2017	8	20	20:06:38	16:06:38
1		40.8722	-71.69217	2017	8	20	20:06:38	16:06:38
1		40.8722	-71.69217	2017	8	20	20:06:38	16:06:38
2	New Jersey	39.18884	-73.56232	2017	8	21	18:00:00	14:00:00
2		39.18884	-73.56232	2017	8	21	18:00:00	14:00:00
2		39.18884	-73.56232	2017	8	21	18:00:00	14:00:00
2		39.18884	-73.56232	2017	8	21	18:00:00	14:00:00
2		39.18884	-73.56232	2017	8	21	18:00:00	14:00:00
2		39.18884	-73.56232	2017	8	21	18:00:00	14:00:00
3	Maryland	37.77692	-74.56461	2017	8	24	15:36:26	11:36:26
3		37.77692	-74.56461	2017	8	24	15:36:26	11:36:26
3		37.77692	-74.56461	2017	8	24	15:36:26	11:36:26
3		37.77692	-74.56461	2017	8	24	15:36:26	11:36:26
3		37.77692	-74.56461	2017	8	24	15:36:26	11:36:26
3		37.77692	-74.56461	2017	8	24	15:36:26	11:36:26
3		37.77692	-74.56461	2017	8	24	15:36:26	11:36:26
5	Georges Bank	41.18026	-67.92692	2017	8	26	16:16:37	12:16:37
5		41.18026	-67.92692	2017	8	26	16:16:37	12:16:37
5		41.18026	-67.92692	2017	8	26	16:16:37	12:16:37
5		41.18026	-67.92692	2017	8	26	16:16:37	12:16:37
5		41.18026	-67.92692	2017	8	26	16:16:37	12:16:37
6	New York	40.75594	-72.19386	2017	8	28	18:26:40	14:26:40
6		40.75594	-72.19386	2017	8	28	18:26:40	14:26:40
6		40.75594	-72.19386	2017	8	28	18:26:40	14:26:40
6		40.75594	-72.19386	2017	8	28	18:26:40	14:26:40
6		40.75594	-72.19386	2017	8	28	18:26:40	14:26:40
4	Off shelf	39.48669	-70.52424	2017	8	25	20:40:52	16:40:52
4		39.48669	-70.52424	2017	8	25	20:40:52	16:40:52
4		39.48669	-70.52424	2017	8	25	20:40:52	16:40:52
4		39.48669	-70.52424	2017	8	25	20:40:52	16:40:52
4		39.48669	-70.52424	2017	8	25	20:40:52	16:40:52
4		39.48669	-70.52424	2017	8	25	20:40:52	16:40:52
4		39.48669	-70.52424	2017	8	25	20:40:52	16:40:52

Table S2- Chlorophyll, DOC, and PAR at sampling stations

Station		Depth	Chlorophyll	DOC	PAR
		m	ug/L	mg/L	uE/m2 s
1	Block Island	4.0	1.03	1.10	1008.4
1		15.1	1.20	1.05	122 .1
1		30.2	0.31	0.96	8.8
1		40.3	0.20	1.10	2.3
1		50.4	0.19	1.02	0.4
1		52.4	nd	nd	0.2
2	New Jersey	3.0	0.71	1.34	1584.6
2		20.2	1.20	0.86	65.3
2		25.2	3.18	0.89	23.8
2		30.2	0.47	0.87	12.2
2		40.3	0.41	0.88	3.3
2		42.3	0.34	nd	2.4
3	Maryland	3.0	0.99	1.45	1595.0
3		10.1	0.83	1.28	248.8
3		17.1	0.47	1.10	67.6
3		24.2	1.00	0.99	25.2
3		34.3	0.12	1.05	4.4
3		50.4	0.08	0.84	0.5
3		54.4	nd	nd	0.3
5	Georges Bank	3.0	2.20	1.16	1195.8
5		8.1	2.12	1.14	395.4
5		30.2	1.97	1.07	2.3
5		40.3	1.74	1.14	0.2
5		49.4	1.66	1.14	0.0
6	New York	4.0	0.51	1.40	928.1
6		12.1	0.67	1.29	233.8
6		18.1	0.60	1.22	81.7
6		30.2	0.45	1.04	13.4
6		40.3	0.57	0.96	2.7
4	Off shelf	3.0	0.08	1.12	643.9
4		25.2	0.12	1.03	148.0
4		54.4	0.45	0.82	30.1
4		100.8	0.02	0.73	2.1
4		160.3	0.00	0.74	0.1
4		320.7	0.00	0.58	0.0
4		2005.8	0.00	0.56	0.0

nd-not determined

Table S3-Dissolved oxygen, nitrite, ammonium, and nitrate at sampling stations

Station		Depth	O_2	NO_2^-	NH_4^+	NO_3^-
		**m**	**uM**	**uM**	**uM**	**uM**
1	Block Island	4.0	257.1	0.246	0.224	bdl
1		15.1	289.9	0.203	0.156	bdl
1		30.2	245.3	0.354	0.872	6.747
1		40.3	214.6	0.416	0.805	5.707
1		50.4	213.5	0.342	0.886	6.582
1		52.4	213.5	nd	nd	nd
2	New Jersey	3.0	228.0	bdl	bdl	bdl
2		20.2	226.0	bdl	bdl	bdl
2		25.2	207.5	0.005	1.149	0.848
2		30.2	207.0	0.083	1.864	3.110
2		40.3	206.5	0.139	2.193	1.607
2		42.3	206.5	0.392	2.075	1.709
3	Maryland	3.0	218.4	bdl	0.037	bdl
3		10.1	216.9	0.103	0.041	1.368
3		17.1	232.2	bdl	0.242	bdl
3		24.2	221.6	bdl	0.263	bdl
3		34.3	204.6	0.455	2.068	2.502
3		50.4	212.0	0.465	2.076	5.029
3		54.4	211.6	nd	nd	nd
5	Georges Bank	3.0	265.2	bdl	0.064	bdl
5		8.1	264.4	bdl	0.071	bdl
5		30.2	262.5	bdl	0.089	bdl
5		40.3	261.9	bdl	0.326	1.616
5		49.4	261.6	0.457	0.181	bdl
6	New York	4.0	249.3	0.035	0.048	nd
6		12.1	260.0	0.240	0.754	nd
6		18.1	238.6	0.566	2.645	nd
6		30.2	211.4	0.356	2.111	nd
6		40.3	203.3	0.336	1.630	nd
4	Off shelf	3.0	214.8	bdl	0.005	nd
4		25.2	233.2	bdl	0.007	nd
4		54.4	185.1	0.045	0.012	nd
4		100.8	176.7	0.275	0.013	nd
4		160.3	189.2	bdl	0.006	nd
4		320.7	133.2	bdl	bdl	nd
4		2005.8	259.8	bdl	0.002	nd

nd-not determined
bdl-below detection limit

Table S4-Flow Cytometry Results

Station		Depth	Avg Nanoeuks	Avg Picoeuks	Avg *Synechococcus*	Avg Bacteria
		m	cells/mL	cells/mL	cells/mL	cells/mL
1	Block Island	4.0	1.3E+03	7.8E+03	1.8E+04	1.3E+06
1		15.1	1.8E+03	2.9E+03	1.2E+05	1.3E+06
1		30.2	3.0E+02	6.8E+02	1.4E+04	5.1E+05
1		40.3	2.2E+02	3.7E+02	5.7E+03	7.1E+05
1		50.4	1.5E+02	4.1E+02	5.7E+03	7.3E+05
1		52.4	nd	nd	nd	nd
2	New Jersey	3.0	5.4E+02	1.0E+03	1.7E+05	5.5E+05
2		20.2	1.1E+03	8.9E+02	6.4E+04	1.3E+06
2		25.2	5.1E+02	5.8E+02	1.7E+04	1.3E+06
2		30.2	4.0E+02	3.8E+02	1.2E+04	6.3E+05
2		40.3	3.7E+02	5.1E+02	1.1E+04	5.7E+05
2		42.3	nd	nd	nd	nd
3	Maryland	3.0	8.9E+02	1.9E+04	1.6E+05	4.2E+06
3		10.1	6.0E+02	1.1E+04	3.6E+05	3.0E+06
3		17.1	4.6E+02	4.6E+03	1.2E+05	1.1E+06
3		24.2	6.2E+02	2.0E+03	4.9E+04	9.1E+05
3		34.3	1.6E+02	7.3E+02	6.3E+03	4.1E+05
3		50.4	1.1E+02	4.8E+02	4.3E+03	4.4E+05
3		54.4	nd	nd	nd	nd
5	Georges Bank	3.0	1.7E+03	3.2E+04	2.5E+04	2.2E+06
5		8.1	1.3E+03	3.3E+04	2.6E+04	2.3E+06
5		30.2	1.1E+03	3.6E+04	2.7E+04	2.0E+06
5		40.3	1.3E+03	3.4E+04	2.8E+04	1.9E+06
5		49.4	1.2E+03	3.4E+04	2.7E+04	1.8E+06
6	New York	4.0	1.3E+03	1.2E+04	4.2E+04	1.2E+06
6		12.1	2.0E+03	1.8E+04	1.0E+05	1.5E+06
6		18.1	1.9E+03	2.1E+03	1.2E+05	1.5E+06
6		30.2	4.3E+02	2.9E+02	1.6E+04	8.4E+05
6		40.3	3.2E+02	1.3E+02	8.6E+03	9.1E+05
4	Off shelf	3.0	2.1E+02	5.6E+02	7.7E+02	6.9E+05
4		25.2	2.5E+02	7.0E+02	3.7E+03	6.3E+05
4		54.4	4.2E+02	4.3E+03	7.8E+03	3.8E+05
4		100.8	bdl	1.9E+02	4.0E+02	1.5E+05
4		160.3	bdl	bdl	2.2E+02	1.3E+05
4		320.7	bdl	bdl	bdl	1.2E+05
4		2005.8	bdl	bdl	bdl	4.1E+04

nd-not determined
bdl-below detection limit

Table S5-R^2 of Pairwise Linear Regressions

R^2 Statistics of Linear Regression Between ROS Measurements and Other Observations		
	Superoxide Concentration	Superoxide Production Rate
Depth	0.03	0.12
Temperature	0.02	0.01
Chlorophyll	0.06	**0.77**
PAR	0.01	0.08
Beam Transmission	0.19	**0.59**
Salinity	0.13	0.23
O_2	0.01	**0.52**
NO_2^-	0.00	0.12
NH_4^+	0.00	0.04
NO_3^-	0.00	0.08
Nanoeukaryotes	0.00	**0.62**
Picoeukaryotes	0.00	**0.74**
Synechococcus	0.00	0.00
Bacteria	0.02	0.27
Dissolved Organic Carbon	0.01	0.16
Superoxide Concentration	--	**0.51**
Superoxide Decay Rate Constant (UFSW)	0.04	**0.62**
Superoxide Decay Rate Constant (AFSW)	0.07	0.35
Contribution of Particles to Total Superoxide Signal (%)	0.00	0.04
Superoxide Production Rate	**0.51**	--

Bold indicates R^2>0.5
